# Investigating the effects of membrane curvature inducing proteins on lipid droplets in *Saccharomyces cerevisiae*

**DOI:** 10.1007/s11033-025-11261-0

**Published:** 2025-11-19

**Authors:** Laura R. K. Niemelä, Isabell Tunn, Alexander D. Frey

**Affiliations:** Department of Bioproducts and Biosystems, Aalto University, Espoo, Finland

**Keywords:** Lipid droplets, Reticulons, Membrane curvature, Endoplasmic reticulum, *Saccharomyces cerevisiae*

## Abstract

**Background:**

Reticulons (Rtn1p, Rtn2p) and Yop1p are proteins shaping the endoplasmic reticulum (ER) membrane curvature their function being to a large degree responsible for molding the membrane curvature in the tubular ER area. Lipid biosynthesis mainly takes place in the tubular parts of the ER and lipid droplets are formed in the tubular ER membrane. Nucleation constitutes the rate-limiting step in lipid droplet formation and is modulated by membrane curvature; specifically increasing membrane curvature lowers the activation energy required for lipid lens nucleation. The aim of this work was to investigate the effects, modifying the ER membrane curvature would have on lipid droplets in *Saccharomyces cerevisiae*.

**Methods and results:**

We created and screened strains overexpressing different combinations of the membrane curvature inducing genes *RTN1*,* RTN2* and *YOP1* and additionally the gene inducing ER membrane expansion, *DGK1*, in four different genetic strain backgrounds. We examined the strains by microscopy and quantified lipid composition by gas chromatography-mass spectrometry. We found that overexpressing membrane curvature inducing proteins decreases the size of the lipid droplets. A reduction in lipid droplet size was accompanied by an apparent increase in their number. We observed that the change in cell size seemed to be partially connected to the strain background. We also noticed altered lipid composition which was linked to the strain backgrounds.

**Conclusions:**

Overexpression of curvature inducing proteins affected lipid droplet size and number, presumably by affecting nucleation. However, the morphological changes of lipid droplets were not accompanied by increased lipid production. Our findings provide a basis for future research on how ER structure modifications influence the lipid droplet dynamics in *S. cerevisiae*.

**Supplementary Information:**

The online version contains supplementary material available at 10.1007/s11033-025-11261-0.

## Introduction

Neutral lipids are stored in lipid droplets (LDs) in eukaryotic cells. LDs are formed from a phospholipid monolayer which originates from the endoplasmic reticulum (ER), where these structures are formed. The phospholipid monolayer carries a variety of proteins on its surface, with a multitude of functions. LDs hold lipid storage function, but they are also operators of cell and ER homeostasis, buffers for lipotoxicity and unfolded proteins [1–4]. LDs were recently recognized as independent cellular organelles and as engineering targets. This diversifies their biological functions and importance, and their applications in biotechnology and medicine. LDs were suggested for example as a scaffold for metabolic pathways and for material applications for example as protectors against oxidative stress or lipotoxicity and for biosensing applications [5–8]. Also, the neutral lipids stored in the LDs are of great interest, as these are of high significance due to applications as raw materials for biofuels and oleochemicals [9–11]. The yeast *Saccharomyces cerevisiae* offers an ideal platform to investigate LDs and their formation in the ER membrane. Even though biosynthesis and storage capacity of lipids are low in the wild type (WT) *S. cerevisiae*, different genetic engineering strategies to increase lipid biosynthesis and lipid accumulation were successfully implemented [9, 12, 13]. Furthermore, *S. cerevisiae* was employed as an organism for the study of LDs and the dynamics of LD formation [14, 15].

The endoplasmic reticulum (ER) is the central hub for lipid biosynthesis and secretory protein biosynthesis. The ER membranes are arranged in the form of sheets and tubular structures. The network of ER sheets and tubules is held together by ER fusion. Essential for the structure of the ER are the membrane curvature inducing reticulon proteins (Rtn1p, Rtn2p) and Yop1p [16]. Sey1p is a mediator of membrane fusion. Lnp1p is a protein that localizes to ER tubular junctions and interacts with Rtn1p, Yop1p, and Sey1p, playing a role in the formation of the tubular ER network [17, 18]. Reticulon proteins and Yop1p shape the tubules by forming wedges in the ER membrane with their hairpin like transmembrane domains and by forming a scaffold on the ER membrane [16, 19, 20]. It was shown that overexpressing genes of membrane curvature inducing proteins leads to an ER with a higher tubule to sheet ratio. Moreover, it was observed that overexpressing reticulon proteins and Yop1p may lead to stiffer tubules as the membrane curvature inducing proteins may oligomerize on the tubule membranes [19, 20]. Also, the increased concentration of membrane curvature inducing proteins on the ER membrane due to overexpression was suggested to reposition other ER membrane proteins [20]. Deleting reticulon proteins and Yop1p was shown to have the opposite effect on the ER structure [16]. Protein biosynthesis mainly happens in the sheet area, and it was previously observed that by expanding the sheets and by increasing the ratio of sheet to tubules it was possible to increase the amount of secreted proteins [21].

Lipid biosynthesis and LD biogenesis mainly take place in the tubular network of the ER. In addition to their lipid storage function LDs protect the cell from lipotoxicity, and against ER stress. It was observed that LDs are formed in the tubular ER membranes. A lipid lens nucleates when a certain concentration of triacylglycerides (TAGs) in the lipid bilayer has been reached, and an expansion of the neutral lipid lens results in budding of a LD [22]. LD formation is affected by the structure of the ER and proteins on the ER membrane which guide the LD formation. Membrane curvature was noted to play an integral role in the formation of LDs. Santinho et al. (2020) showed that an increasing membrane curvature in tubules decreases the concentration of TAGs needed for LD nucleation, due to the higher chemical potential of TAGs in tubules compared to flat sheets [22]. The activation energy needed for nucleation may be lowered by the presence of following structural factors like increased membrane curvature by local deformation of the membrane, increased curvature by curvature inducing proteins, and lowered nucleation energy by proteins and/or lipids which interact with TAGs and steer them towards nucleation [22, 23]. The molecular structures of lipids exhibit different curvatures which effect the membrane structure and LD formation process [15, 24]. The surface tension of the ER membrane was observed to control LD formation and size, where decreasing tension favors the emergence of LDs. A factor that controls the surface tension of the ER membrane is its phospholipid composition [24]. ER membrane density refers to how tightly membrane components – such as lipids and proteins – are packed within the bilayer. The phospholipid composition strongly influences both membrane density and membrane curvature. Differences in phospholipid headgroup structure lead to variations in local lipid packing, while the fatty acyl chain composition particularly the degree of saturation, further modulates membrane density. Unsaturated fatty acids with cis bonds introduce kinks that reduce packing efficiency, whereas saturated fatty acids are more linear and allow tighter packing within the membrane [25, 26].

Other ER membrane associated proteins influence the formation, emergence and stabilization of lipid droplets. In *S. cerevisiae* a protein complex Sei1/Ldb16 is centrally involved in the generation of LDs [14, 27]. In mammalian and plant cells Seipin occurs without a partner. Seipin (Sei1) is concentrated in ER tubules, with LD budding occurring at Seipin enriched sites [14, 22, 28]. Seipin also facilitates and directs the lipid flow to the LDs regulating their growth [29, 30]. Santinho et al. (2020) showed in human and plant cell lines that LDs may also bud in the absence of Sei1p [22]. It was however noted that these LDs are formed in an unstable manner and are heterogenous in their appearance [22, 29]. Perilipin is a membrane protein involved in stabilizing the LD and it also collaborates with other membrane proteins in guiding the formation of the LDs [31]. Finally, fat storage-inducing transmembrane proteins 2 (FIT2) that are found in abundance in areas of LD formation, are responsible for the emergence of the LDs from the ER membrane [15]. *SCS3* and *YFT2* are *S. cerevisiae* homologs to the mammalian *FIT2* and also showed to be important for the ER stress response [32].

LDs contribute to the restoration of the cellular homeostasis by different mechanisms [33]. It has been observed that LDs may remove excess fatty acids, phospholipids and unfolded proteins from the ER and by doing so alleviate stress [1, 4]. It was observed that LDs are essential for maintaining ER homeostasis and buffering lipid induced stress [1, 4, 34]. Excess fatty acids may be stored as TAGs in LDs to protect the cell from potential cytotoxic effects [1, 4, 35]. Vevea et al. (2015) demonstrated that *S. cerevisiae* responds to chronic lipid imbalance resulting from inhibition of phosphatidylcholine (PC) synthesis by channeling phospholipids into TAGs, storing them in LDs, and ultimately trafficking these to the vacuole for degradation. Lipid imbalance was shown to activate the UPR, which in turn promoted LD biogenesis. Vevea et al. (2015) also observed that during lipid stress unfolded proteins were stored into LDs which were associated with polyubiquitinated proteins and Kar2p, an ER chaperone, and transported to the vacuole where they were degraded. These findings highlighted the involvement of LDs in proteostatic quality control and the preservation of lipid and cellular homeostasis.

Lipids are accumulated in the form of droplets and in this research the goal was to modify the ER morphology of *S. cerevisiae* as nucleation of LDs may be augmented with increasing curvature and this was expected to translate into changes in the formation of LDs. To achieve this, we overexpressed genes of membrane curvature inducing proteins *RTN1*, *RTN2*, and *YOP1* and additionally *DGK1* a gene which has been connected to the expansion of the nuclear ER in different combinations in *S. cerevisiae* [36]. We included four different strain backgrounds and investigated the effects of *RTN1*, *RTN2*, *YOP1* and *DGK1* expression on cell and LD morphology, UPR, and lipid content and composition. We observed morphological changes of the LDs and cell size. We found differences in lipid composition which were mostly connected to the strain background and an altered UPR.

## Materials and methods

### Generation of yeast strains

All yeast strains used in this study (Supplementary Information Table S1) are based on the laboratory strain W303α, designated later as wild type (WT). Genes *RTN1*,* RTN2*,* YOP1*, and *DGK1* were amplified from genomic DNA and cloned into yeast shuttle vectors pRS416, pRS415, and pRS413 containing a TEF promoter. Oligonucleotides used in this study are listed in Supplementary Information Table S2. All plasmids used in this work are listed in Supplementary Information Table S3. Plasmids were transformed into the yeast strains according to the lithium acetate method by Gietz and Schiestl (2007) [37]. Gene deletions were created according to the protocol by Hegemann and Heick (2011) by transforming knockout cassettes with loxP sites created by PCR from the plasmids pUG6 and pUG74 carrying marker genes into yeast. The selection markers were removed by expressing Cre-recombinase from the plasmid pSH47 [38].

Strains YLN126, YLN127, and YLN128 with the GFP based UPR reporter were created using the pDEP17 plasmid [39] containing 4 repeats of UPREs upstream of a minimal promotor and a NdegY-GFP sequence. The pDEP17 plasmid was linearized with EcoRV and integrated into the genome into the *TRP1* locus. The YMR024 reporter strain was created previously [40].

### Yeast cultures

Yeast strains without plasmids were grown in YPD medium. Selective synthetic drop-out (SD) -medium (0.67% yeast nitrogen base (YNB) without amino acids, 0.6 g/L amino acid mix, and 20 g/L glucose as a carbon source) lacking histidine, uracil, and leucine was employed for strains carrying plasmids.

For lipid production yeast cells were grown over night in 5 mL synthetic drop-out medium lacking histidine, uracil, and leucine. 50 ml shake flask cultures with fresh selective media were inoculated with overnight pre-cultures and cultured for 24 h at 30 °C and 220 rpm shaking after which cells were harvested and inoculated in 50 ml yeast carbon base (YCB) -medium (1.7 g/L YNB, 20 g/L glucose, tryptophane 0.2 g/L, methionine 0.2 g/L) with 50 mM sodium phosphate buffer (pH 6.5). Shake flask cultures were continued for 48 h at 30 °C and 220 rpm shaking. Cells were harvested by centrifuging the samples 10 min and washing the cell pellet with sterile water and repeating centrifugation. Subsequently the cells were broken by adding 500 µL sterile water and 500 µL acid washed glass beads (425–600 μm) to the cell pellets and by vortexing for 45 min (Cell disruptor genie) at 4 °C. After which the cell suspension was moved to a new tube and stored at −20 °C before drying.

### Yeast microtiter plate cultivations

Growth curves were recorded by conducting continuous absorbance measurements from microtiter plates. Precultures were grown in synthetic dropout medium lacking uracil, histidine, and leucine for 24 h at 30°C. For the determination of growth curves cultures were inoculated in clear round bottom 96-well plates to an OD_600_ of 0.1 in 100 µL of synthetic dropout medium without uracil, histidine, and leucine. Absorbance measurements were conducted in 15 min intervals for 48 h at 30°C and shaking in double orbital mode (807 rpm 1 mm) with a Cytation 3 microplate spectrophotometer (BioTek, Winooski, USA).

We conducted fluorescence signal measurements for investigating the UPR in 96- well black well plates. Precultures were grown in synthetic dropout medium without uracil, histidine, and leucine at 30 °C and 220 rpm shaking. The fluorescence signal was determined in microtiter plate cultivations in synthetic dropout medium without uracil, histidine, and leucine and in experiments with yeast carbon base media under nitrogen starvation. When the yeast carbon base media was used, cultures were inoculated to an OD_600_ of 4 (corresponding to 0.4 in the plate reader) in 100 µL of media. UPR was induced with 2.5 µM DTT or 0.1% Tween-80 to increase fatty acid stress. The fluorescence signal (excitation 485/20, emission 528/20) was measured in 15 min intervals for 48 h at 30 °C and shaking in double orbital mode (807 rpm 1 mm) with a Cytation 3 microplate spectrophotometer (BioTek, Winooski, USA).

### Lipid content determination

Frozen yeast samples were freeze dried for a week with a freeze dryer (Christ alpha 2–4), after which the dry mass of the samples was determined. Cellular lipids were determined by gas chromatography-mass spectrometry (GC-MS). Sample preparation was carried out by direct saponification and methylation as described by Suutari et al. (1990) [41]. Heptadecanoic acid methyl ester (40 mg/mL) dissolved in hexane was used as an internal standard and 5 µL of the internal standard was added to each sample with a syringe. The dried sample was suspended in 1 mL of saponification reagent 3.7 M NaOH in 49% methanol. Samples were flushed with N_2_ and mixed by vortexing. Samples were kept at 100 °C for 5 min, vortexed and returned to a 100 °C water bath for 25 min. Samples were cooled down to room temperature in a water bath and 4 mL methylation reagent (3.3 M HCl in 48% Methanol) was added. The sample was mixed by vortexing, held at 80 °C for 10 min, and cooled down to room temperature. Fatty acid methyl esters were extracted in 1.5 mL hexane/methyl tert-butyl ether solution (1:1). The samples were shaken for 10 min at 500 rpm with a laboratory rotator—model G2 (New Brunswick Scientific Co. Inc.) and the lower phase was removed with a Pasteur pipette. The samples were washed with 3 mL 0.3 M NaOH and shaken for 5 min at 500 rpm with the laboratory rotator—model G2 after which the samples were centrifuged at 3214 rcf for 20 min and the upper phase was collected [41]. The sample was dried with anhydrous Na_2_SO_4_, flushed with N_2_ and analyzed with GC-MS. The major fatty acids were identified from their peak retention times. The samples were analyzed with a Shimadzu GC-MS with optic 4 with a HP1MS column (60 m x 0.25 mm x 0.25 μm). The following settings were used for the measurements. Column flow rate was 0.22 mL/min and the total flow rate to the detector 5.4 mL/min. The column inlet pressure was 20.0 kPa. Linear velocity was 12.5 cm/s and septum purge flow rate 3.0 mL/min. The split ratio was 1:10. The column oven temperature was programmed from 120°C to 250 °C. The ion source temperature was 200 °C and the interface temperature was 250 °C. Solvent cut time was 4 min, event time 0.3 s, scan speed 1666, start m/z 35 and end m/z 500. Peaks were determined with Shimadzu Labsolutions data analysis program.

### Fluorescence microscopy

Nile Red staining of the lipid droplets was conducted as essentially described in Rostron et al. (2017) [42]. The Nile Red stock was dissolved in dimethyl sulfoxide (DMSO). 250 µL of culture was collected. Nile Red dye was added in the dark at room temperature to a final concentration of 5 µg/mL. The cell cultures were incubated for 5 min in the dark after which they were washed four times with phosphate buffered saline (PBS). The cell pellets were resuspended in PBS for imaging. For each yeast strain about 100 cells (59–153) with their LDs from 4 to 8 images were evaluated from the samples harvested 48 h after cultivation in YCB media. For each yeast strain 38 to 268 LDs were evaluated from the samples harvested 8 h after cultivation in YCB media. 3,3’-Dihexyloxacarbocyanine iodide (DiOC6(3)) was used to stain the ER according to Koning et al. (1993) [43]. A 10 mg/mL DiOC6(3) stock dissolved in ethanol was used to prepare the final staining concentration of 10 µg DiOC6(3)/OD_600_ of cells [43]. Strains were grown in synthetic dropout medium without uracil, histidine, and leucine. 1 OD_600_ of cells was harvested, spun down and resuspended in 1 mL PBS. 1 µL of DiOC6(3) stock solution was added to the resuspended cells.

Image acquisition of the Nile Red or DiOC6(3) stained yeast cells was done using a Zeiss Axio Observer Z1/7 microscope with a 100 × 1,4 oil Plan-Apochromat objective and a 1,6x tube lens. Both stains were excited at 470 nm with a LED light source at 20% intensity. A 61 HE GFP/HcRed filter set (Zeiss) was employed. The emitted light was collected with a 30 ms exposure time when imaging the Nile Red stained cells. The emitted light of the DiOC6(3) stained yeast cells was collected with a 10 ms exposure time. Zeiss Software Zen blue 3.2 was used for image acquisition and Zeiss Software Zen lite 3.8 was used for image analysis.

### Data–analysis

Data-analysis and visualization were done using the GraphPad Prism 10.0 software. Ordinary one-way ANOVA followed by Tukey’s multiple comparisons method was applied for analyzing experimental results. The curve fitting was performed by nonlinear regression with the Gompertz growth function. The maximum growth rate and the maximum UPR induction rate (the slope of the GFP level) were determined from the inflection point. The maximum UPR induction rate was calculated using Microsoft Excel and the maximum growth rate was calculated by the BioTek Gen5 (2.9 version) software.

## Results

We investigated the effects of membrane curvature inducing proteins (Rtn1p, Rtn2p, Yop1p) expressed alone or in different combinations on LD formation in four strain backgrounds: W303α (WT), *Δopi1*, *Δtgl3Δtgl4* and *Δopi1Δtgl3Δtgl4*. The rationale to select these genotypes was to minimize the interference of lipid accumulation by lipid mobilization from LDs. This was implemented by deletion of the lipase genes *TGL3* and *TGL4*, which encode LD localized lipases catalyzing TAG hydrolysis. A more detailed description of the Tgl3p and Tgl4p roles can be found in Supplementary Information Table S4. Deletion of the *OPI1* gene was intended to promote expansion of the ER membranes and potentially increase the surface area available for LD budding. Furthermore, the *OPI1* gene deletion leads to constitutively active expression of phospholipid biosynthetic genes. Moreover, simultaneous overexpression of Rtn1p was reported to modify the expanded membranes into tubules [44]. The *Δopi1Δtgl3Δtgl4* strain background was created with the assumption that this strain might combine beneficial traits of the *Δopi1* and *Δtgl3Δtgl4* strain backgrounds. Genes of the membrane curvature inducing proteins were expressed in all eight possible combinations in the selected strain backgrounds. Also, Dgk1p, which is localized to the ER membrane and participates in lipid metabolism and which overexpression leads to ER membrane expansion (especially nuclear ER expansion), was transformed in the 4 different strain backgrounds [36]. The purpose of expressing Dgk1p was to analyze the effect of ER expansion in the different strain backgrounds. Dgk1p catalyzes the formation of phosphatidic acid (PA) from diacylglycerol (DAG) and counteracts phosphatidic acid phosphatase. PA is an important intermediate in the formation of various phospholipids [36]. *DGK1* transformation in the *Δopi1Δtgl3Δtgl4* strain background was however unsuccessful, hence only three strains overexpressing *DGK1* were created. Altogether, we created 35 strains to be screened in their LD formation features. The expected outcomes of the modifications that we introduced, are listed in Supplementary Information Table S5.

### Growth phenotype

**Fig. 1:**
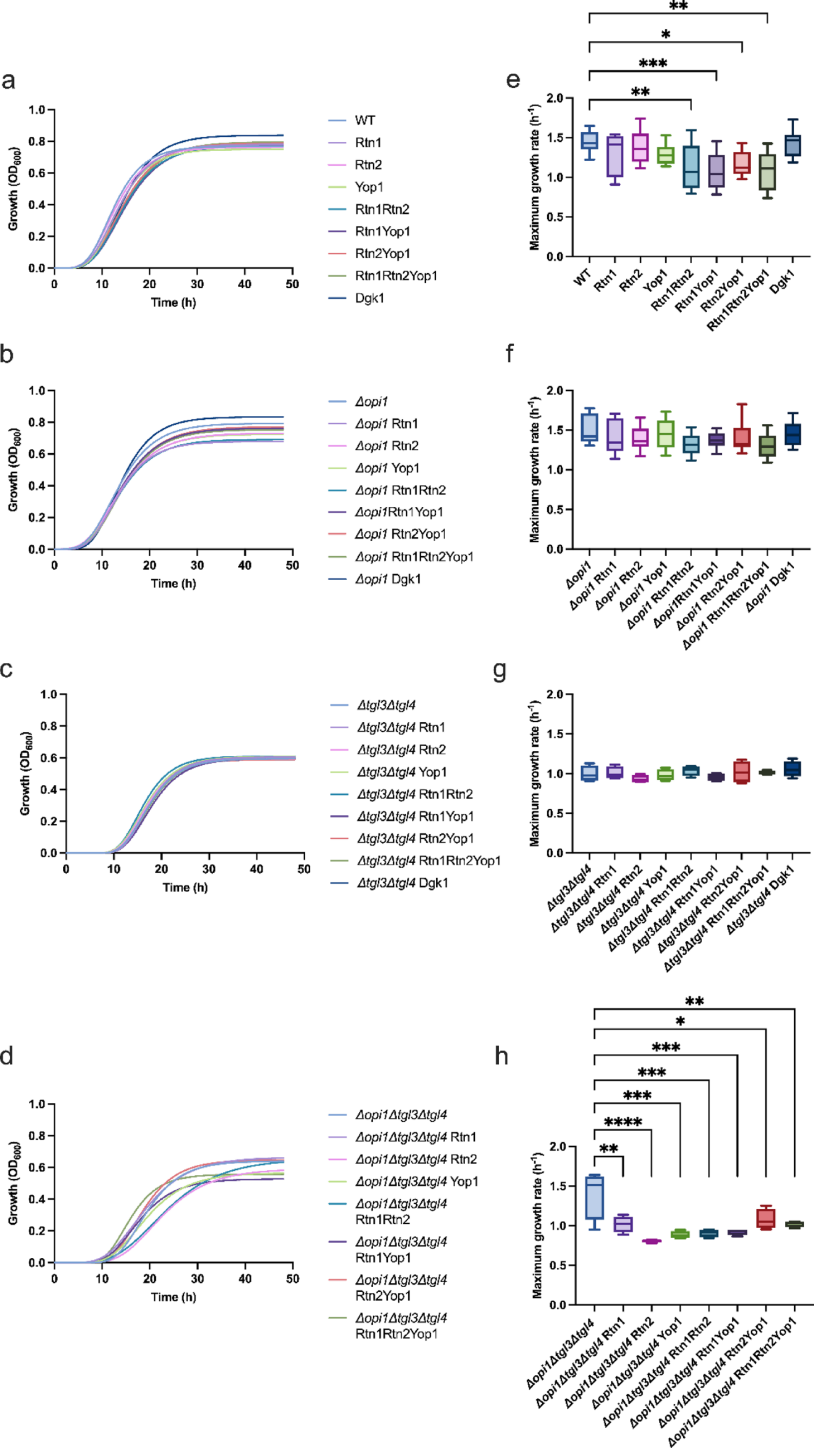
Growth phenotype of *S. cerevisiae* strains overexpressing genes of membrane curvature inducing proteins and their corresponding controls. Strains were grown in 96-well microtiter plates at 30°C and shaking at 807 rpm (1 mm) in SD-UHL media and the OD_600_ was measured every 15 min for 48 h. Panels **a**–**d** present the growth curves of different strains sorted according to their genetic backgrounds, **e**–**h** show the maximum growth rates of different strains sorted according to their genetic backgrounds. The strain backgrounds are (**a**) and (**e**) WT; (**b**) and (**f**) *Δopi1*; (**c**) and (**g**) *Δtgl3Δtgl4*: (**d**) and (**h**) *Δopi1Δtgl3Δtgl4*. 4–15 replicates per strain were used. The boxplots show the distribution of the biological replicates of the determined maximum growth rates. Whiskers extend to the minimum and maximum values and the central line represents the median. P-values were calculated by pairwise comparison to the corresponding control *P < 0.05; **P < 0.01; ***P < 0.001, ****P < 0.0001

We investigated the growth phenotype of the control strain and modified strains as we were interested in seeing if overexpressing membrane curvature inducing genes would influence growth. The gene deletions in the strain background generally effected the growth as shown in Fig. 1. The *Δopi1* effected growth only mildly (Fig. 1c, d): only final cell density was affected, maximum growth rate seemed unaffected in comparison to the WT strain background. *Δtgl3Δtgl4* (Fig. 1e, f) and *Δopi1Δtgl3Δtgl4* (Fig. 1g, h) both diminished the final cell density and maximum growth rate. The overexpression of membrane curvature inducing genes did not seem to decrease the final OD_600_ of cultures in the WT strain background in comparison to the control strain. However, the growth rate was affected in strains in which two or more membrane curvature inducing proteins were overexpressed, these presented a significantly decreased growth rate in comparison to the control. In the *Δopi1* background the overexpression of reticulons and Yop1p had an effect on final cell density but seemingly not on growth rate. In the *Δtgl3Δtgl4* strains the overexpression of membrane curvature inducing proteins did not seem to have an effect on final cell density or growth rate, whereas in the *Δopi1Δtgl3Δtgl4* strains both the final cell density and the growth rate were affected by the overexpression of reticulons and Yop1p. All of the overexpression strains presented a significantly decreased growth rate in comparison to the corresponding control (Fig. 1).

### The modified strains presented changes in cell size and ER structure

**Fig. 2:**
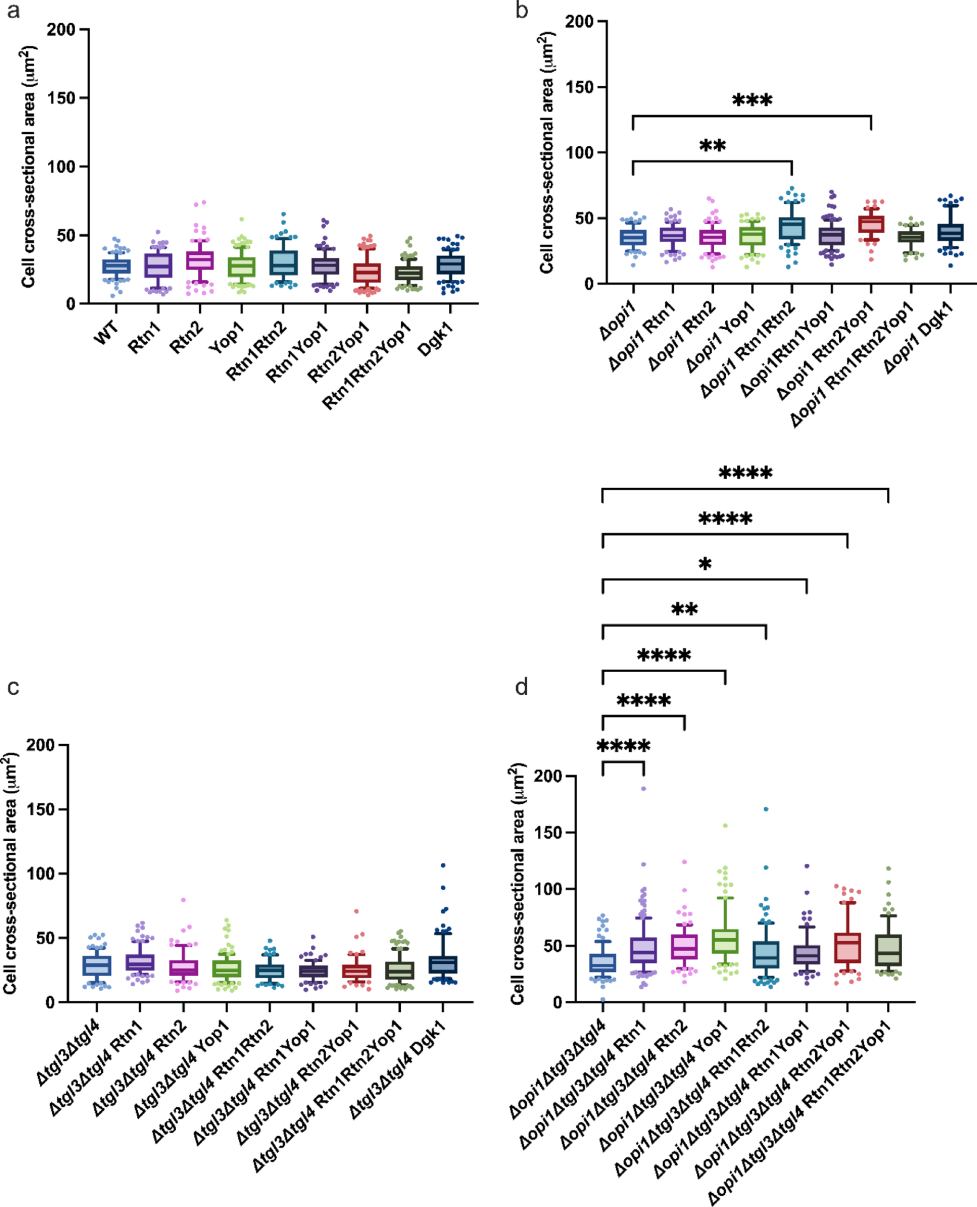
The cell cross-sectional area of *S. cerevisiae* strains overexpressing genes of membrane curvature inducing proteins and their corresponding controls. The cross-sectional area was determined by microscopy from about 100 cells (59–153) per strain. Panels **a**–**d** show the cell cross-sectional areas of different strain backgrounds: (**a**) WT (**b**) *Δopi1* (**c**) *Δtgl3Δtgl4* (**d**) *Δopi1Δtgl3Δtgl4*. The boxplots show the distribution of the replicates of the determined cell cross-sectional areas. Whiskers extend to the 10th and 90th percentile values and the central line represents the median. P-values were calculated by pairwise comparison to the corresponding control *P < 0.05; **P < 0.01; ***P < 0.001, ****: *P* < 0.0001

We evaluated about 100 cells per strain (59–153) by microscopy to understand the morphological effects induced by our modifications as presented in Fig. 2 and in the Supplementary Information Tables S4a, b. The strains with the *Δopi1* showed a higher cross-sectional area in comparison to strains which still harbored the *OPI1* gene. In comparison to the WT strain background the strains with the *Δopi1* background presented a 1.27–1.67-fold higher average cross-sectional area. When comparing the average cross-sectional area of the overexpression strains to the *Δopi1* control strain, only the Rtn1Rtn2 (YLN075) strain and Rtn2Yop1 (YLN077) showed a significant change in average cross-sectional area. The *Δopi1* Rtn1Rtn2 strain presented 1.26-fold average cell cross-sectional area in comparison to the corresponding control and the *Δopi1* Rtn2Yop1 strain presented 1.31-fold average cell cross-sectional area in comparison to the corresponding control. The *Δopi1Δtgl3Δtgl4* strains presented a broader distribution of the measured cross-sectional area than the other strains. These strains also showed the greatest cross-sectional area overall (Fig. 2d). In comparison to the unmodified control strain the average cross-sectional area was 1.31-fold to 2.13-fold higher in these strains. When the average cross-sectional area of the overexpression strains was compared to the *Δopi1Δtgl3Δtgl4* control strain the average cell cross-sectional area was 1.23-fold to 1.62-fold higher depending on the strain. The strains of the other two strain backgrounds did not show significant changes in cell size. The effect of the *Δopi1* expanding the ER membranes has been observed previously by Schuck et al. (2009) and in our previous work [21] we observed not only the effect of the *Δopi1* expanding the ER membranes, but we established the effect the *Δopi1* has on expanding the cell size. The effect of overexpressing membrane curvature inducing proteins was not as clear as the effect of the strain background on the cell cross-sectional area. The overexpression of reticulons and/or Yop1p did not seem to have an effect on cell cross-sectional area in the strains which still carried the *OPI1* gene. It seemed as if in the *Δopi1* and *Δopi1Δtgl3Δtgl4* strains the cell cross-sectional area increased with increasing overexpression of membrane curvature inducing proteins and then did not increase further by the overexpression of all three genes.

We stained the strains overexpressing all three membrane curvature inducing proteins and the corresponding controls with DiOC6(3) (10 µg/mL) according to Koning et al. (1993) to investigate the architecture of the ER [43]. We chose the triple overexpression strains as it was expected that these strains would present the most changes and the corresponding controls to compare them to. All of the triple overexpression strains seemed to present a denser tubular ER web than the corresponding controls. As the cells in the *Δopi1Δtgl3Δtgl4* background were bigger in size compared to the cells in the other strain backgrounds, observing overexpression induced changes was easier in this strain background (Supplementary Information Fig. S1). Cells from the triple overexpression strains exhibited a higher abundance of membrane structures compared to the corresponding controls.

### Overexpression of membrane curvature inducing genes effected lipid droplet size and shape

**Fig. 3:**
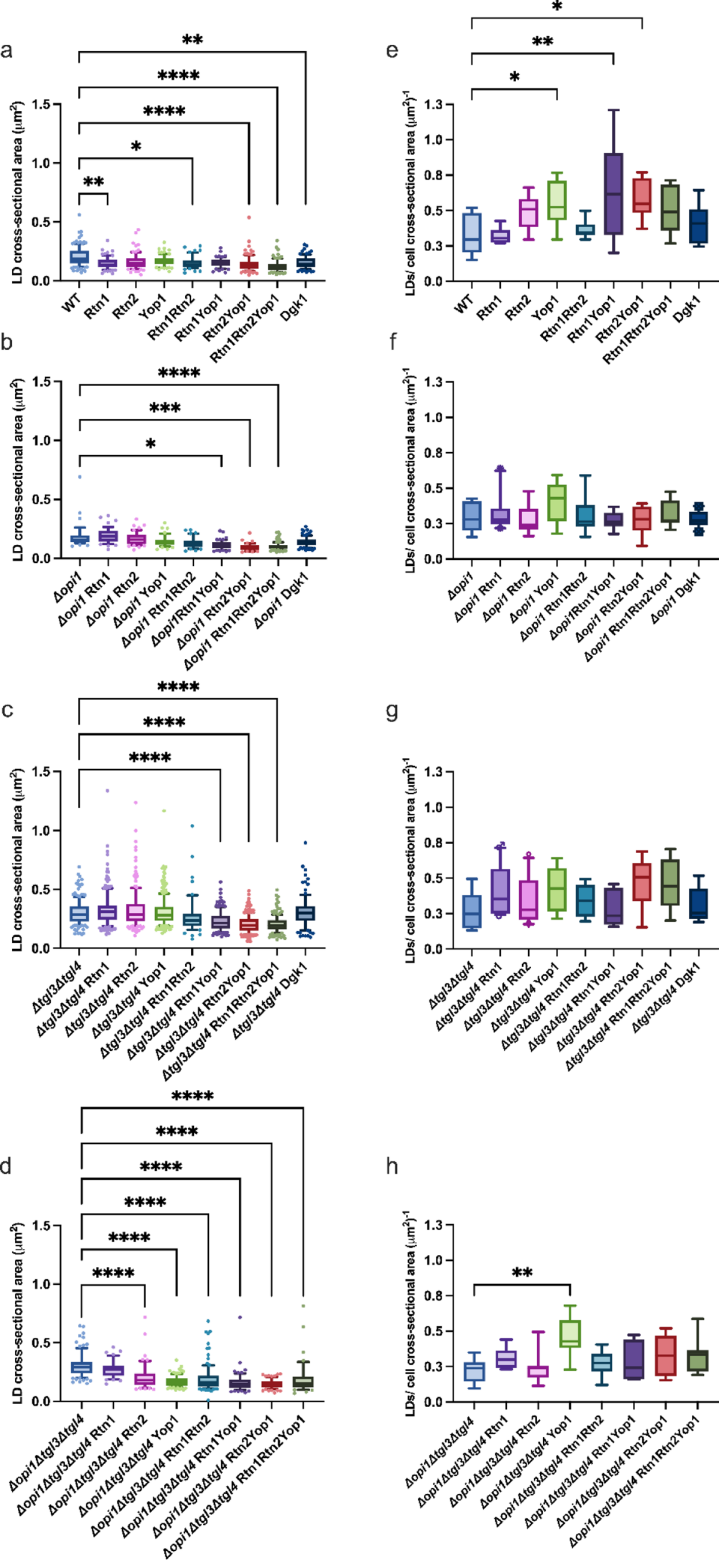
LD cross-sectional area and LDs per cell cross-sectional area of *S. cerevisiae* strains overexpressing genes of membrane curvature inducing proteins and their corresponding controls. The cross-sectional area was determined by microscopy from 38–268 LDs per strain. Panels **a**–**d** show the LD cross-sectional areas of different strain backgrounds: (**a**) WT (**b**) *Δopi1* (**c**) *Δtgl3Δtgl4* (**d**) *Δopi1Δtgl3Δtgl4*. LDs per cell cross-sectional area were determined by microscopy from 6–10 cells per strain. Panels **a**–**d** show the LD cross-sectional areas of different strain backgrounds: (**e**) WT (**f**) *Δopi1* (**g**) *Δtgl3Δtgl4* (**h**) *Δopi1Δtgl3Δtgl4*. The boxplots show the distribution of the replicates of the determined LD cross-sectional areas or LDs per cell cross-sectional area. Whiskers extend to the 10th and 90th percentile values and the central line represents the median. P-values were calculated by pairwise comparison to the corresponding control *P < 0.05; **P < 0.01; ***P < 0.001, ****P < 0.0001

We evaluated the size of the formed LDs after 8 h of cultivation in YCB media. Per strain, 38–268 LDs were assessed from microscopy pictures and LD size was expressed as cross-sectional area. Generally, the LD size seemed to decrease with increasing overexpression of membrane curvature inducing proteins as presented in Fig. 3 and in Supplementary Information Table S4a, b. In the WT strain background, the average LD cross-sectional area varied from 0.60-fold to 0.84-fold in comparison to the control. The triple overexpression strain Rtn1Rtn2Yop1 (YLN067) and the double overexpression strain Rtn2Yop1 (YLN066) showed the greatest difference in LD cross-sectional area in comparison to the WT control strain (YLN060) as shown in Fig. 3a. Both strains had formed LDs which presented a significantly smaller cross-sectional area than the LDs of the control strain. In the *Δopi1* strain background the average LD cross-sectional area varied from 1.05-fold to 0.51-fold in comparison to the corresponding control (YLN079). The triple overexpression Rtn1Rtn2Yop1 (YLN078) strain and two double overexpression strains Rtn2Yop1 (YLN077), and Rtn1Yop1 (YLN076) presented a significant shrinkage in LD cross-sectional area in comparison to the control (YLN079) as shown in Fig. 3b.

When the overexpression strains in the *Δtgl3Δtgl4* background were compared to their respective control, the double and triple overexpression strains presented a decreased LD cross-sectional area (Fig. 3c). The strains Rtn1Yop1 (YLN113), Rtn2Yop1 (YLN114), and Rtn1Rtn2Yop1 (YLN115) showed a significantly decreased average LD cross-sectional area 0.77-fold to 0.67-fold depending on the strain. When the overexpression strains in the *Δopi1Δtgl3Δtgl4* background were compared to their respective control, all but YLN119, Rtn1p overexpressing strain, presented significantly decreased LD cross-sectional area (Fig. 3d). In comparison to the respective control strain the average cross-sectional area of the overexpression strains was 0.90-fold to 0.49-fold depending on the strain.

Both double lipase knockout strain backgrounds presented a generally larger LD cross-sectional area in comparison to the WT strain. The two strain backgrounds with *TGL3* and *TGL4* gene deletions were observed to show after 8 h of culturing under nitrogen starvation some giant LDs. Under for example stressful situations the fatty acid composition of membranes especially the ER and the LDs may be affected, which may influence the fusion of LDs [24, 45–47]. 

We quantified the number of LDs per cell cross-sectional area after 8 h of cultivation under nitrogen-limited conditions. In the WT background, LD numbers in the overexpression strains ranged from 0.98-fold to 1.95-fold relative to the WT control. A significant increase in LD abundance was observed in strains overexpressing *RTN1*, *RTN1YOP1*, and *RTN2YOP1* (Fig. 3e). In the *Δopi1* background, the overexpression strains exhibited a greater average cell cross-sectional area; however, this increase exceeded the rise in LD numbers resulting in 0.70- to 1.03-fold LD numbers per cell cross-sectional area compared with the corresponding control (Fig. 3f). In the *Δtgl3Δtgl4* background, the overexpression strains displayed 1.04- to 1.74- fold LD numbers per cell cross-sectional area relative to the corresponding control, though none of these changes were statistically significant (Fig. 3g). In the *Δopi1Δtgl3Δtgl4* background, LD numbers per cell cross-sectional area increased by 1.08-fold to 2.01-fold depending on the strain, compared to the corresponding control. A significant increase was detected only in the strain overexpressing *YOP1* (Fig. 3h).

**Fig. 4:**
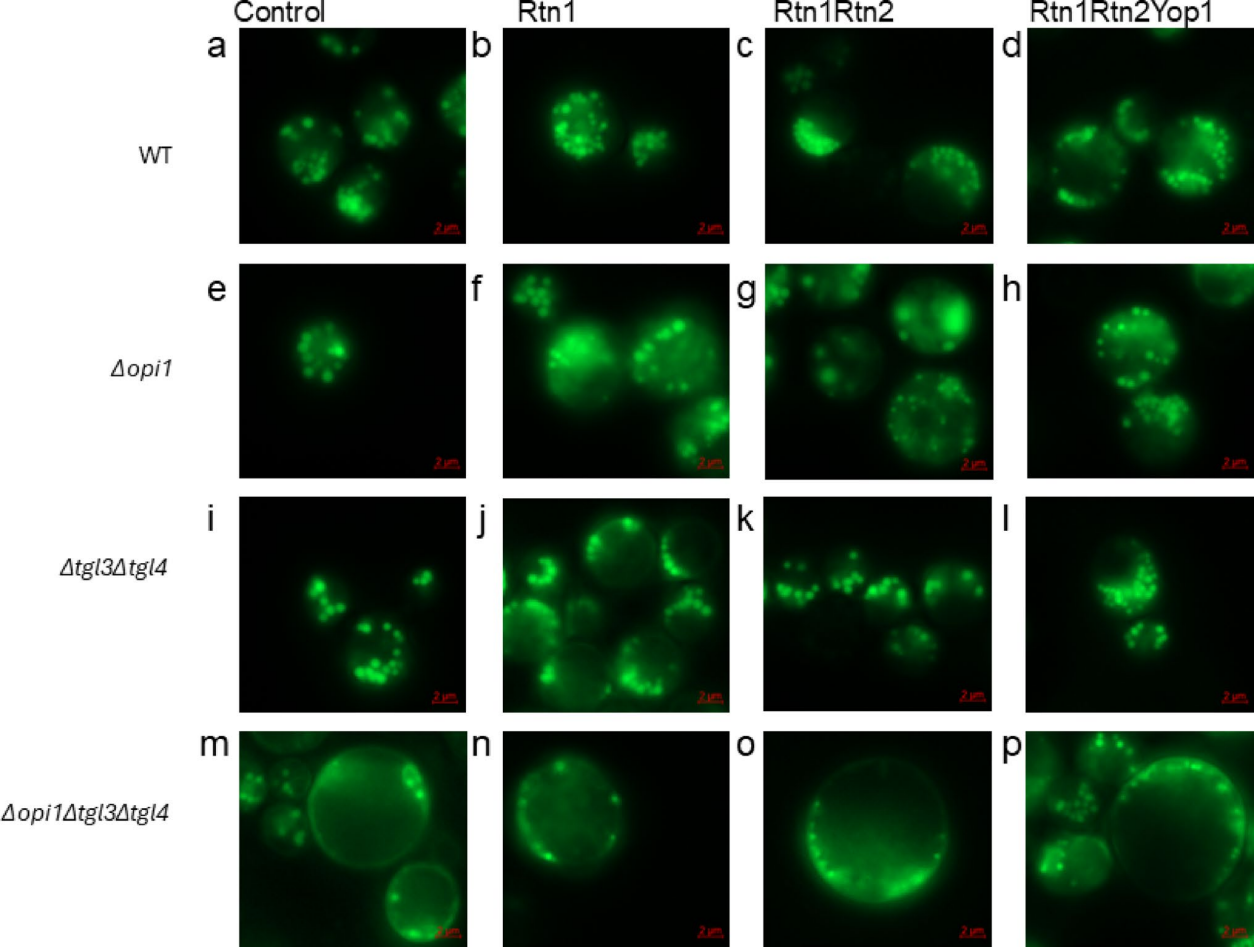
Nile Red stained LDs in *S. cerevisiae* strains overexpressing membrane curvature inducing proteins and their corresponding controls. Panels a – d present selected strains in the WT background: (**a**) WT control; (**b**) Rtn1; (**c**) Rtn1Rtn2; (**d**) Rtn1Rtn2Yop1. Panels **e**–**h** present selected strains in the *Δopi1* background: (**e**) *Δopi1* control; (**f**) *Δopi1* Rtn1; (**g**) *Δopi1* Rtn1Rtn2; (**h**) *Δopi1* Rtn1Rtn2Yop1. Panels **i**–**l** present selected strains in the *Δtgl3Δtgl4* background: (**i**) *Δtgl3Δtgl4* control; (**j**) *Δtgl3Δtgl4* Rtn1; (**k**) *Δtgl3Δtgl4* Rtn1Rtn2; (**l**) *Δtgl3Δtgl4* Rtn1Rtn2Yop1. Panels **m**–**p** present selected strains in the *Δopi1Δtgl3Δtgl4* background: (**m**) *Δopi1Δtgl3Δtgl4* control; (**n**) *Δopi1Δtgl3Δtgl4* Rtn1; (**o**) *Δopi1Δtgl3Δtgl4* Rtn1Rtn2; (**p**) *Δopi1Δtgl3Δtgl4* Rtn1Rtn2Yop1. Scale bar: 2 μm

When cells were inspected under the microscope after 48 h of culturing under nitrogen starvation a substantially higher number of LDs was observed. Part of these occurred as huddled masses in the cells. Figure 4 shows a comparison of Nile Red stained LDs in a control strain, single overexpression strain, double overexpression strain, and triple overexpression strain in all the strain backgrounds after 48 h of culturing (the corresponding brightfield images are presented in the Supplementary Information Fig. S2). Generally, LDs of the strains overexpressing membrane curvature inducing proteins seemed smaller as observed with the samples which were harvested after 8 h of culturing, but after 48 h of culturing it was observed that there seemed to be more LDs which had grown, or undergone fusion and appeared larger or were irregular in their form.

### ER membrane modifications led to changes in lipid droplet fatty acid composition

**Fig. 5:**
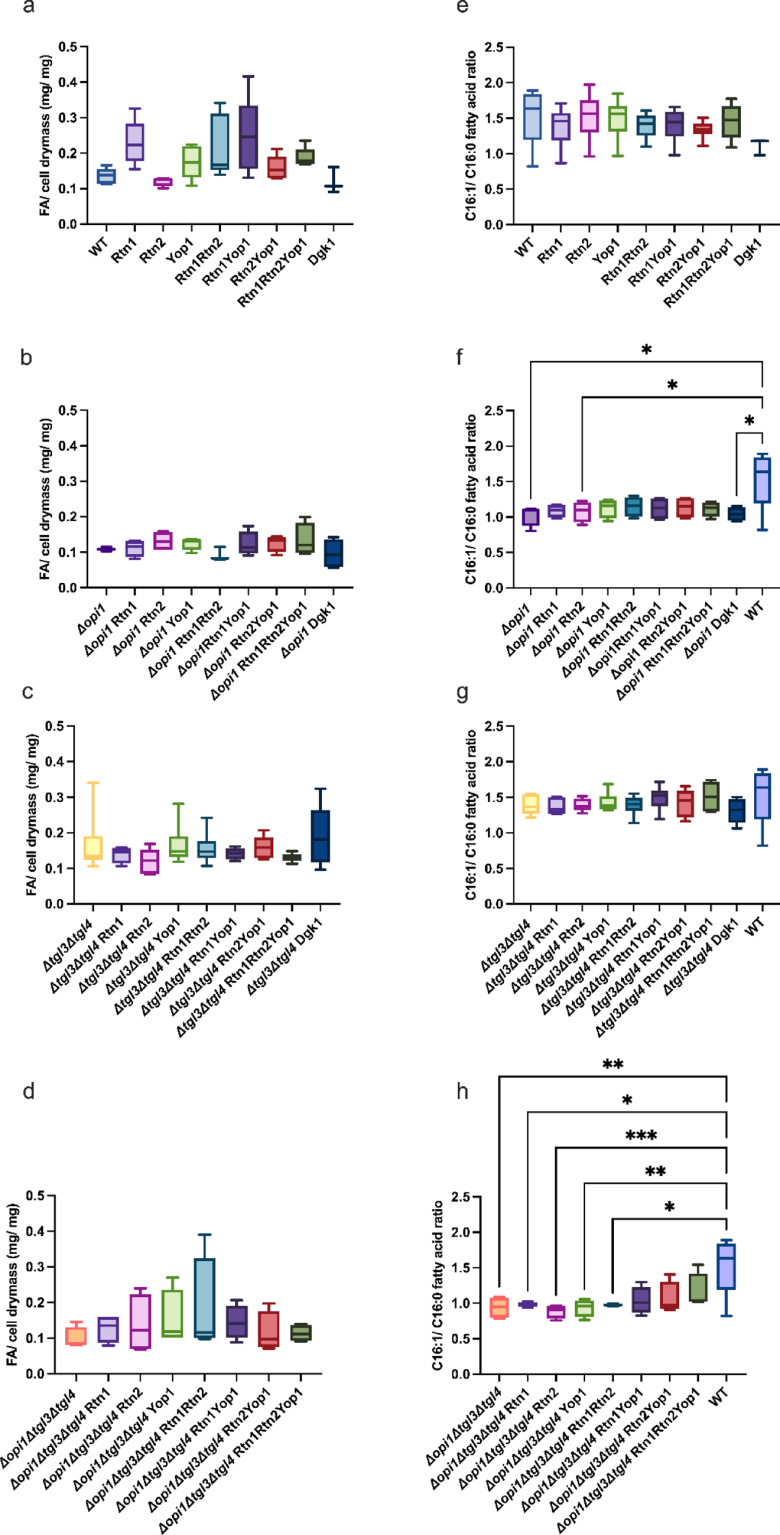
Lipid content and composition of *S. cerevisiae* strains overexpressing membrane curvature inducing proteins and their corresponding controls. Extracted fatty acids (mg) per cell dry mass (mg) were determined with GC-MS and are shown in panels **a**–**d** sorted to strain background. The determined C16:1 to C16:0 fatty acid ratio in the samples is presented in the panels **e**–**h** sorted to strain background. The strain backgrounds are (**a**) and (**e**) WT; (**b**) and (**f**) *Δopi1*; (**c**) and (**g**) *Δtgl3Δtgl4*: (**d**) and (**h**) *Δopi1Δtgl3Δtgl4*. 4 to 6 biological replicates were used for the measurements. Boxplots show the distribution of the biological replicates of the determined fatty acids per cell dry mass (**a**–**d**) and C16:1 to C16:0 fatty acid ratio (**e**–**h**). Whiskers extend to the minimum and maximum values and the central line represents the median. P-values were calculated by pairwise comparison to the corresponding control and WT control strain. *P < 0.05; **P < 0.01; ***P < 0.001, ****P < 0.0001

As the implemented ER membrane modifications were expected to increase the curvature of the tubular ER membrane, which may increase lipid droplet nucleation, we investigated how this would affect lipid content and composition. Samples for lipid analysis were harvested after 48 h of cultivation under nitrogen starvation. The total average lipid content (Fig. 5a and Supplementary Information Table S4a) in the strains with the WT background varied from 0.89-fold to 1.81-fold in comparison to the control strain. Only strains overexpressing Rtn2 (YLN062) and Dgk1 (YLN096) presented lower average lipid content in comparison to the control strain (YLN060). In the *Δopi1* strain background the average lipid content varied from 0.85-fold to 1.23-fold in comparison to the corresponding control strain (Fig. 5b and Supplementary Information Table S4a). Strains in the *Δtgl3Δtgl4* background presented 0.74-fold to 1.17-fold average lipid content in comparison to the corresponding control (YLN108) - the overexpression strains either presented similar average lipid content or even lower lipid content than the corresponding control strain (Fig. 5c and Supplementary Information Table S4b). The overexpression strains in the *Δopi1Δtgl3Δtgl4* background showed 1.15-fold to 1.82-fold average lipid content in comparison to the corresponding control (YLN118) as presented in Fig. 5d and Supplementary Information Table S4b. None of the average lipid content differences were significant.

The fatty acid compositions of the samples are presented in Supplementary Information Tables S5a, b. Generally, it seemed that the strains with the *Δopi1* showed a slightly higher saturated fatty acid content in comparison to the strains carrying the *OPI1* gene. This was especially evident in the altered ratio of monounsaturated (C16:1) to saturated (C16:0) fatty acids both comprising 16-carbon chains. The ratio of C16:1 to C16:0 (Fig. 5b) of the strains in the WT background ranged from 1.33 to 1.48 as an exception the strain overexpressing Dgk1p, which in all of its traits resembled the strains of the *Δopi1* background. The Dgk1p overexpression strain showed a 1.11 ratio of C16:1 to C16:0. The strains in the *Δtgl3Δtgl4* background showed a C16:1 to C16:0 ratio from 1.31 to 1.51. Here as well the strain overexpressing Dgk1p presented the lowest ratio. Strains in the *Δopi1* background displayed C16:1 to C16:0 ratios from 1.02 to 1.14 and strains in the *Δopi1Δtgl3Δtgl4* background presented C16:1 to C16:0 ratios from 0.89 to 1.15. Although C12:0 and C14:0 show a low percentage in the fatty acid composition some interesting differences could be observed in the share of these fatty acids in the different strain backgrounds. In the WT background strains C12:0 holds 1% of fatty acid composition, like in the *Δtgl3Δtgl4* background strains, but in the *Δopi1* strains its share increased to 1.40–1.79% of the fatty acid composition and interestingly in the *Δopi1Δtgl3Δtgl4* strains a slightly lower share from 0.63 to 0.94% was observed. The C14:0 show in the strains with the *Δopi1* background a slight increase. As previously pointed out in the WT strain background the Dgk1p overexpression strain is exceptional and resembles the *Δopi1* strains, showing a 1.75% C12:0 of the fatty acid composition and a 3.35% of C14:0.

According to statistical analysis none of the overexpression combination strains improved lipid content per cell dry mass significantly in comparison to their corresponding control. Many of the strains produced a high variance in the results. Especially the overexpression strains were observed to be highly susceptible to outside factors in their lipid production capacity. But significant changes were observed in the lipid composition according to strain background. Generally, strains with the *Δopi1* seemed to present higher levels of saturated fatty acids. Strains with the *Δopi1* showed a significantly lower C16:1 to C16:0 ratio in comparison to the WT strains.

### Shaping the ER membrane effected the unfolded protein response

As we modified the ER membrane curvature, we were interested in the effects on the unfolded protein response (UPR). Our motivation to investigate the UPR was enhanced by the observation that when the ER sheets are expanded the UPR diminishes [21]. We studied the UPR of the modified and control strains. The UPR receptor Ire1p is located in the ER membrane. It is activated by accumulating unfolded proteins and lipid bilayer stress (aberrant lipid composition of the ER membrane) [45, 48, 49]. It has been observed that with increased membrane curvature and narrower tubular space the location of membrane proteins like the Ire1p might be affected [20]. This also may affect Ire1p in its actions as it oligomerizes for activation. A denser packing of the proteins may lead to easier activation of the UPR and/or increased UPR levels.

To investigate the UPR in the modified strains we conducted a time course analysis and induced the UPR under nitrogen starvation with a final concentration of 2.5 mM DTT to increase the amount of unfolded proteins and with a final concentration of 0.1% Tween-80 to induce stress, we also studied the strains under nitrogen starvation without any additional induction methods. A GFP based UPR responsive transcriptional reporter was integrated into the genome of the strains for detecting the UPR. We employed an additional control in all of the strain backgrounds, expressing the induced *HAC1*^*i*^ gene that is constitutively active from a plasmid.

We investigated the UPR under nitrogen starvation (under lipid producing conditions), the measurements were started at OD_600_ of 4 (corresponding to an OD_600_ read of 0.4 on the plate reader). It was expected that under these conditions there would be little growth as the external glucose would be steered into glycolysis and fatty acid synthesis and the resulting fatty acids would be stored as neutral lipids in LDs. In the comparison of the results, we took into consideration the GFP level at the starting point of measurements at induction with DTT and Tween-80.

**Fig. 6:**
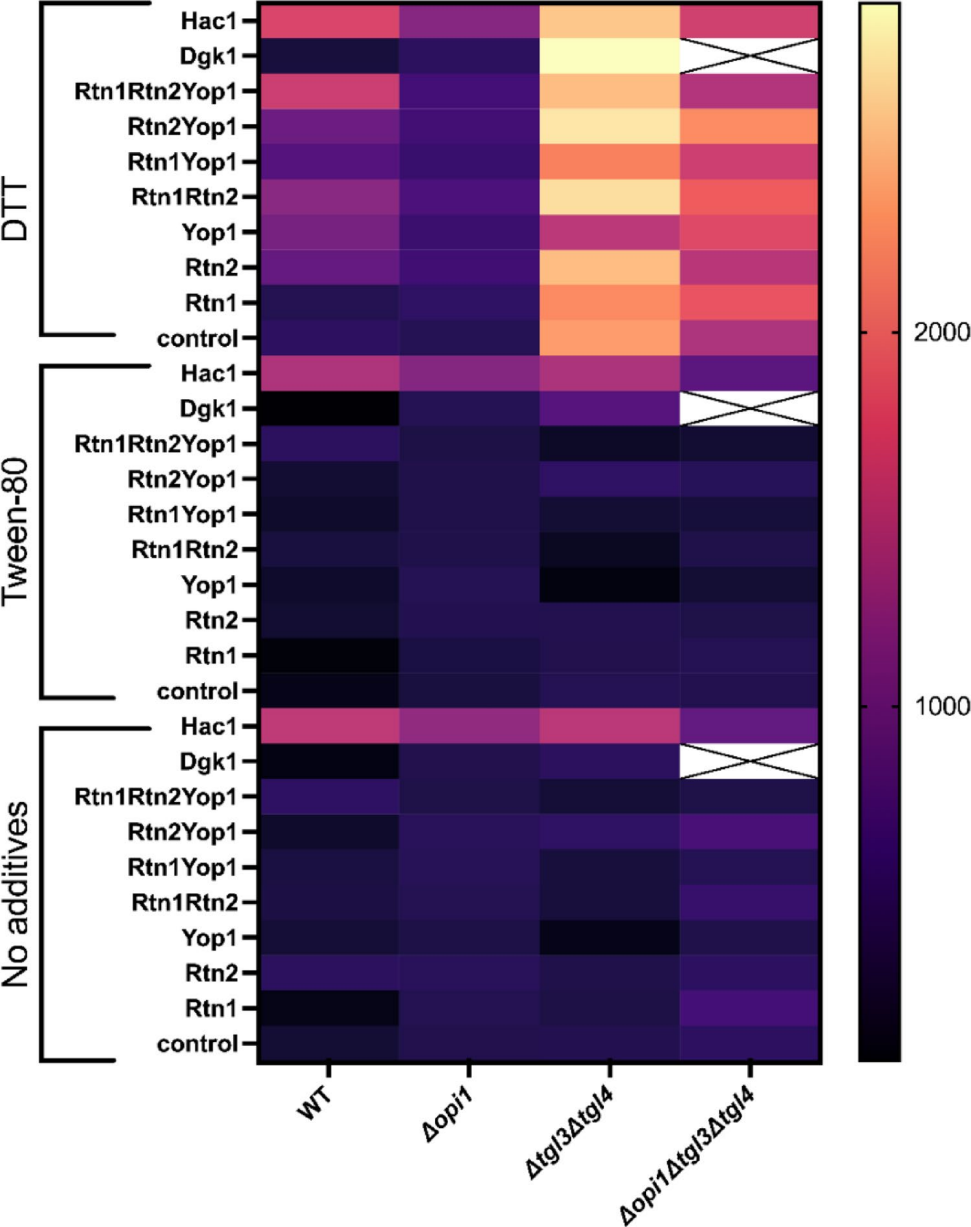
Maximum UPR induction rates of the modified strains and corresponding controls under the influence of DTT, Tween-80, and without any UPR inducing additives. The heatmap colour scale represents the maximum UPR induction rate. Six biological replicates were used to determine the maximum UPR induction rates. The white panels which are crossed through contain no data

When comparing maximum GFP fluorescence levels which were reached during the experiment in the WT strain background, there seemed to be an increased GFP expression (corresponding to a higher UPR) with increasing overexpression of membrane curvature inducing genes under all conditions (Supplementary Information Fig. S3a, e, i, m, q, u). The control strain expressing *HAC1*^*i*^ presented under all conditions the highest GFP levels (4.6–6.1-fold in comparison to the control). When inducing UPR with DTT the strains overexpressing one protein reached maximum fluorescence values of 0.8–2.1. Yop1p reached a fold of 2.1 in comparison to the control whereas the other three strains overexpressing a single protein presented lower values 0.8–1.9-fold in comparison to the control, the strain overexpressing Dgk1p showing the lowest GFP levels in comparison to the control (0.8-fold). The strains overexpressing two proteins presented 1.6–2.3-fold maximum GFP levels in comparison to the control. The strain overexpressing both reticulon proteins and Yop1p showed 3.2-fold determined maximum GFP levels in comparison to the control. The determined maximum UPR induction rates at the exponential phase of the time course analysis curves showed similar results as the maximum GFP level results: the highest maximum UPR induction rates were observed with the strain overexpressing both reticulon proteins and Yop1p and the control strain expressing the induced Hac1p (Fig. 6 and Supplementary Information Fig. S3a, e). The cultures with Tween-80 addition and the cultures without any additions presented similar results, only the GFP level reached was generally lower, except for the strain expressing *HAC*^*i*^, which presented the highest GFP levels compared to the control in the non-induced conditions and the lowest in the DTT induced conditions, still showing the highest GFP levels of all strains. Supplementary Information Tables S8a, b, c, d present the maximum GFP level, the increase in GFP level from the time point of induction, and fold.

In the *Δtgl3Δtgl4* background strains, under DTT induced conditions, the single membrane curvature inducing protein overexpressing strains presented 0.6–1.0-fold GFP levels in comparison to the corresponding control. The Dgk1p overexpressing strain showed 1.35-fold GFP levels in comparison to the corresponding control. The strains which were overexpressing two membrane curvature inducing proteins presented 1.0–1.6-fold GFP levels in comparison to the corresponding control. The strain overexpressing both reticulon proteins and Yop1p showed a 1.1-fold GFP level and the Hac1p expressing strain a 1.4-fold GFP level in comparison to the corresponding control. When the WT strain was compared to the *Δtgl3Δtgl4* strain it showed 0.3-fold GFP levels and the WT strain seemed to show a higher UPR induction rate. Interestingly, the Dgk1p overexpressing strain presented the second highest GFP levels in comparison to the corresponding control in the *Δtgl3Δtgl4* strain after the *HAC*^*i*^ expressing strain in all conditions. There is not a clear effect which would link the overexpression of the reticulon proteins and Yop1p to the UPR induction rate in all of the strain backgrounds like in the unmodified WT strain background. The only parameters with a clear effect on the maximum UPR induction rate are the inducers. The maximum UPR induction rate was higher in the cultures which were induced with *HAC*^*i*^ or DTT. With DTT induction the single overexpression strains in the *Δopi1* background presented maximum GFP levels 1.2–1.3-fold in comparison to the corresponding control strain and the overexpression strains with two membrane curvature inducing proteins being overexpressed presented about 1.4-fold maximum GFP levels in comparison to the corresponding control. The strain overexpressing both reticulon proteins and Yop1p presented 1.3-fold GFP levels in comparison to the corresponding control and the strain expressing Dgk1p presented 1.2-fold GFP levels in comparison to the corresponding control. The strain expressing Hac1p presented the highest GFP levels in the *Δopi1* background (2.6-fold in comparison to the corresponding control). Thus, all strains present higher GFP levels in comparison to the corresponding control, and the strains overexpressing two membrane curvature inducing proteins show the highest GFP levels in comparison to the control in all conditions (excluding the *HAC1*^*i*^ expressing control strain). With the strains in the *Δopi1* background there is no clear strain specific effect on the maximum UPR induction rates, only the induction method causes a visible significant effect. The strains in which Hac1p is constitutively expressed and activates the UPR presents the highest maximum UPR induction rates, also induction with DTT showed the highest maximum UPR induction rates in comparison to Tween-80 and no induction. In the *Δopi1Δtgl3Δtgl4* strain background generally only the DTT induced cells presented higher GFP levels in comparison to the corresponding control. The *HAC1*^*i*^ expressing control strain showed 1.1–1.2-fold GFP levels in comparison to the corresponding control in the different conditions. For the *Δopi1Δtgl3Δtgl4* background strains as well as for the *Δtgl3Δtgl4* background strains in a slightly lesser extent it could be observed that at the point when the measurements were started the GFP levels of the overexpression strains were already higher than in the corresponding control strain. The strains were grown for 48 h in SD medium lacking histidine, uracil and leucin before inoculating the strains into YCB media lacking nitrogen and starting measurements. This could be a sign of the effect the overexpression of the membrane curvature inducing proteins has on the UPR. Generally, one could argue that the increase in GFP levels with increasing overexpression of membrane curvature inducing proteins could only be a consequence of increased protein expression stress.

## Discussion

In this work we set out to engineer the membrane curvature of the ER to modify LD formation. The connection of ER membrane structure, membrane curvature inducing proteins (Rtn1p, Rtn2p, Yop1p), and LDs seemed intriguing to us. We were interested in the changes we could induce on the LDs by modifying the membrane curvature: if this would have an effect on the composition of the LDs, the morphology or if this would increase the formation of LDs and thereby their accumulation. We also investigated the effect of deleting two lipase genes (*TGL3* and *TGL4*) in combination of overexpressing membrane curvature inducing genes with the assumption that this may have an effect on lipid accumulation as in the absence of the lipases lipid mobilization from the LDs would be diminished. We were also interested in the effect of the *Δopi1* in combination to overexpressing genes of membrane curvature inducing proteins, as the *Δopi1* leads to constitutive expression of phospholipid biosynthetic genes and ER membrane expansion and it was previously observed that the expanded sheets of an *Δopi1* strain may be changed into tubular structures when Rtn1p is overexpressed [16, 44]. Schuck et al. (2009) proposed that the *Δopi1* leading to phospholipid biosynthetic genes being constitutively overexpressed would provide a higher capacity for building a tubular network by overexpressing genes of membrane curvature inducing proteins [44].

We discovered that with increasing overexpression of membrane curvature inducing proteins the LDs became significantly smaller, and there seemed to be a larger number of smaller LDs in the cells. Santinho et al. (2020) observed that a smaller angle of the ER membrane of the tubules leads to decreased activation energy needed in LD formation: a smaller concentration of TAGs is needed for the lipid lens to form [22]. Our observations of smaller LDs in increased numbers in strains overexpressing membrane curvature inducing proteins aligns with the results of Santinho et al. (2020). If a smaller concentration of TAGs is needed for the lipid lens to form, then smaller LDs probably emerge in increased amounts. We also concluded that LDs have a tendency to fuse or become larger with longer culturing time, which seemed to apply especially to the strains which were overexpressing membrane curvature inducing proteins. This may be due to ER stress, and UPR induced changes of the phospholipid composition of the ER membrane which then affects the composition of the LD membrane as it originates from the ER membrane. These observations seem to be supported by the findings of Fei et al. (2011). They proposed that increased PA levels facilitate LD fusion and that both PA and PE, due to their cone-shaped geometry and membrane-curvature-modulating properties, play a critical role in this context [47]. Also, the protein composition or localization of ER membrane proteins may be affected by the overexpression of reticulon proteins and Yop1p, as these may oligomerize on the ER membrane as they mold the tubules and thus delocalize other ER membrane proteins [20].

Generally, the control strains in the *Δtgl3Δtgl4* and *Δopi1Δtgl3Δtgl4* strain background showed larger LD cross-sectional areas. The *Δopi1Δtgl3Δtgl4* control strain presented similar LD cross-sectional area to the *Δtgl3Δtgl4* control strain, but strains overexpressing reticulon proteins and/or Yop1p presented smaller LD cross-sectional areas in the *Δopi1Δtgl3Δtgl4* strain. As phospholipid biosynthetic genes are constitutively overexpressed due to the *Δopi1* this could be a reason for the significantly smaller LDs. Even though we deleted the lipases Tgl3p and Tgl4p, the LDs still carried functional Tgl5p, Ayr1p and Ldh1p lipases on the LDs which may mobilize lipids from the LDs for phospholipid synthesis and membrane expansion [50–53]. Interestingly Vevea et al. (2015) suggested that an excess in phospholipid biosynthesis would be stored as TAGs in LDs [4]. We, however, observed in strains lacking the *OPI1* gene predominantly an increase in cell size. Overexpression of membrane curvature inducing genes resulted in a decrease in LD size. We attributed this effect to increased incorporation of phospholipids into the ER membranes leading to expansion of the membrane.

Since we deleted the genes *TGL3* and *TGL4*, we anticipated a partial restriction in the mobilization of TAGs from the LDs [50, 51]. Cells were cultivated in a two-step process: the first step aimed to promote biomass accumulation, while the second step induced lipid accumulation under nitrogen-limiting conditions. Under nitrogen starvation the activity of the cytosolic lipases decreases, but the vacuolar lipase activity increases, as nitrogen starvation has been observed to induce LD microautophagy (lipophagy) [54]. LDs are taken into the vacuole and degraded; this limits the benefits of the lipase deletions. We also observed a growth defect in the strains with the *TGL3* and *TGL4* deletions. In addition to the nitrogen starvation induced lipophagy, lipid/ER stress induces LD microautophagy which is a separate process from the nitrogen starvation induced LD microautophagy [4, 34, 54]. Both processes presumably have an effect on the number of detected LDs in the cells and the determined lipid amount.

We observed that the modified cells showed changes in cross-sectional area. These changes in cell cross-sectional area were partially connected to the strain background. The strains overexpressing membrane curvature inducing proteins in strain backgrounds with the *Δopi1* presented significant changes in cross-sectional area in comparison to the corresponding control. As it has been previously observed *Δopi1* induces membrane expansion as phospholipid biosynthetic genes are constitutively expressed [44, 55] which might be a contributing factor here: with increased phospholipid production, followed by membrane expansion due to deposition of increased amounts of phospholipids in the membranes the cells may slowly expand. The cells of the *Δopi1Δtgl3Δtgl4* strains, especially the cells of the strains overexpressing membrane curvature inducing proteins, showed a significantly larger cross-sectional area in comparison to the corresponding control and to the WT. We found that the lipid composition in the strain backgrounds lacking the *OPI1* differs from the strains which still carry the *OPI1* gene. The strains with the *Δopi1* in the strain background presented a higher percentage of saturated fatty acids, especially the ratio of C16:1 to C16:0 fatty acids was significantly lower in most of the strains lacking the *OPI1* gene compared to the WT. Saturated and unsaturated fatty acids effect the structure in the phospholipid membrane, as phospholipids carrying saturated fatty acids enable a tighter packing of the phospholipids in the membrane leading to a more rigid membrane structure. The double bonds of the unsaturated fatty acids with cis bonds need more space in the phospholipid membrane leading to increased fluidity of the membrane, whereas unsaturated fatty acids with trans bonds behave similarly as saturated fatty acids [25, 26].

The effects of membrane curvature-inducing proteins on increased cell cross-sectional area were only detected in strain backgrounds lacking *OPI1*, indicating that the genetic background plays a critical role in this phenotype. The observed increase in cell cross-sectional area is likely driven by the constitutive expression of phospholipid biosynthetic genes in *Δopi1* strains [44], resulting in membrane expansion. These strains also exhibited elevated levels of saturated fatty acids, which promote tighter lipid packing within the phospholipid bilayer and consequently increased membrane rigidity. Overexpression of membrane curvature inducing proteins likely further enhances membrane rigidity through oligomerization of these proteins on the ER membrane [20]. We propose that the increased rigidity of the ER membrane necessitates greater cellular volume, as rigid membranes require more space for proper accommodation compared to fluid membranes.

The overexpression of membrane curvature inducing proteins seemed to increase lipid production in comparison to the corresponding controls variably depending on the strain background. There was no significant increase detected, but the strains in the WT background and in the *Δopi1Δtgl3Δtgl4* seemed to show higher average lipid production with increased overexpression of membrane curvature inducing proteins in comparison to their corresponding controls, whereas in the *Δopi1* strains the effect was mild to moderate and in the *Δtgl3Δtgl4* strains there did not seem to be an improvement. Moreover, the susceptibility of the strains to external factors can affect the produced lipid concentration, which is reflected in the high variance in the results. The increased average lipid production in the overexpression strains might be a combined effect of increased membrane curvature, an increased UPR, which has probably led to increased gene expression of lipid biosynthetic genes and membrane expansion.

Increased ER membrane curvature and smaller tubular lumina are bound to have an effect on the UPR, as it has been suggested that with a shrinking tubular lumen diameter there might be changes in the location of ER membrane proteins like Ire1p which is the UPR sensor in *S. cerevisiae* [20]. The phospholipid composition is also important for stress sensing, as membrane bilayer stress may also induce the UPR [45, 48, 49]. Especially in the *Δopi1* strains the fatty acid composition was altered which may affect the induction of the UPR. Also, changes in the phospholipid composition may change the structure of the membranes: membranes containing higher amounts of PA and PE (lower amounts of PC) have been observed to affect the organization of the membrane, changing for example the curvature of the membranes [47].

The strains overexpressing membrane curvature inducing proteins presented an altered UPR. Ishiwata-Kimata et al. (2025) found in their studies that by controlled UPR inducing conditions the ER expanded, and lipid production increased [56]. We suspected that the increased UPR in our strains resulted from changed positioning of membrane proteins causing altered protein interactions, protein overexpression stress, and lipid/membrane stress. The *Δopi1* strains with constitutively overexpressed phospholipid biosynthetic genes presented an altered fatty acid composition as already mentioned but compared to the WT background there was no improvement detected in lipid production. In the *Δopi1Δtgl3Δtgl4* strains we expected a restricted TAG mobilization from the LDs. But as in the *Δopi1* strains it seems that the constitutive expression of phospholipid biosynthetic genes led to increased membrane synthesis/membrane expansion in comparison to the WT cells. The cells were also significantly larger in comparison to the WT strains. The UPR of the overexpression strains seemed increased when induced with DTT but there seemed to be no increased lipid production in comparison to the wt strains. Oleate has been previously used as an external stimuli/stressor which enhances LD formation. This has been also suggested to stimulate vacuolar uptake of LDs [54]. Lipid and ER stress induced autophagy represent distinct pathways from nitrogen starvation induced autophagy, yet both may also account for the lower-than-expected lipid levels, as we had anticipated that the strains would show increased lipid accumulation compared to the control strains [4, 34, 54].

## Conclusions

We investigated the relationship between ER membrane curvature inducing proteins and LDs and our analysis primarily revealed effects on LD and cell morphology. Overexpression of membrane curvature inducing proteins led to a significant reduction in LD size. This observation aligns with our expectations, as increased ER membrane curvature – resulting from the overexpression of these proteins – is known to reduce ER tubule diameter and influence the concentration at which LD nucleation occurs [20, 22]. In selected strains we also observed a significant increase in LD number per cell cross-sectional area. In most cases, strains that produced enlarged cells did not display a corresponding increase in LD numbers per cell area, likely due to the relative expansion of the cell size. This increased cell area in *Δopi1* strains is consistent with the constitutive expression of phospholipid biosynthetic genes in this background. We propose that the increased membrane rigidity caused by a possibly higher saturated fatty acyl group content in *Δopi1* strains, together with the additional rigidity from reticulon protein and/or Yop1p overexpression, contributes to the expanded cell cross-sectional area observed in the strains.

Finally, we found that overexpression of membrane curvature inducing proteins intensified the UPR under nitrogen limited conditions, with the magnitude of this effect varying across strain backgrounds.

Further studies are required to determine whether, and to what extent, the induced modifications alter the protein composition of the ER membrane and LDs, and how such changes may impact other processes, such as lipid droplet fusion in yeast. Our findings have potential implications for future studies involving LDs and ER membrane associated processes or applications.

## Supplementary Information

Below is the link to the electronic supplementary material.

Supplementary Material 1

## Data Availability

All data pertaining to the findings of this study are available within this paper. Unprocessed raw data is provided upon request. All materials are shared with academic institutions upon request and execution of a material transfer agreement.
