# Supplementary material for: Investigating the effects of membrane curvature inducing proteins on lipid droplets in Saccharomyces cerevisiae
Source: Mol Biol Rep. 2025 Nov 19;53(1):102. doi: 10.1007/s11033-025-11261-0 (PMC12630273; doi:10.1007/s11033-025-11261-0)
Supplement: Supplementary file 1 — Supplementary Material 1 [file 11033_2025_11261_MOESM1_ESM.docx]

**Supplementary data**

**Investigating the effects of membrane curvature inducing proteins on lipid droplets in *Saccharomyces cerevisiae***

**Laura R. K. Niemelä*, Isabell Tunn and Alexander D. Frey**

Aalto University, Department of Bioproducts and Biosystems, Espoo, Finland

*Corresponding author to whom proofs and preprints should be addressed to:

Laura Niemelä,

laura.niemela@aalto.fi

[https://orcid.org/0009-0009-4382-5997](https://eur01.safelinks.protection.outlook.com/?url=https%3A%2F%2Forcid.org%2F0009-0009-4382-5997&data=05%7C02%7Claura.niemela%40aalto.fi%7C95b4cae0bccf42efa28508ddc6db188e%7Cae1a772440414462a6dc538cb199707e%7C1%7C0%7C638885366525991072%7CUnknown%7CTWFpbGZsb3d8eyJFbXB0eU1hcGkiOnRydWUsIlYiOiIwLjAuMDAwMCIsIlAiOiJXaW4zMiIsIkFOIjoiTWFpbCIsIldUIjoyfQ%3D%3D%7C0%7C%7C%7C&sdata=iVn2vgHFGVHVnHCxvvphJ1hN1ObcTbXb%2Fuzq4MAGYwA%3D&reserved=0)

Supplementary Table S1a. *Saccharomyces cerevisiae* strains used in this study.

| Name | Genotype or genetic modifications | Reference |
| --- | --- | --- |
| W303α | MATα *leu2-3,112 trp1-1 can1-100 ura3-1 ade2-1 his3-11,15* | ATCC 208353 |
| YEK014 | W303α; *Δopi1::loxP* | [1] |
| YMR24 | W303α; *trp1-1::TRP1::NdegY-GFP* | [2] |
| YLN107 | W303α; *Δtgl3::loxP Δtgl4::loxP* | This study |
| YLN117 | YEK014; *Δtgl3::loxP Δtgl4::loxP* | This study |
| YLN126 | YEK014; *trp1-1::TRP1::NdegY-GFP* | This study |
| YLN127 | YLN107; *trp1-1::TRP1::NdegY-GFP* | This study |
| YLN128 | YLN117; *trp1-1::TRP1::NdegY-GFP* | This study |
| YLN060 | W303α + pEK7 + pEK17 + pEK18 | This study |
| YLN061 | W303α + pEK36C + pEK7 + pEK18 | This study |
| YLN062 | W303α + pEK17 + pLN14 + pEK18 | This study |
| YLN063 | W303α + pEK17 + pEK7 + pLN15 | This study |
| YLN064 | W303α + pEK36c + pLN14 + pEK18 | This study |
| YLN065 | W303α + pEK36c + pEK7 + pLN15 | This study |
| YLN066 | W303α + pEK17 + pLN14 + pLN15 | This study |
| YLN067 | W303α + pEK36c + pLN14 + pLN15 | This study |
| YLN072 | YEK014 + pEK36C + pEK7 + pEK18 | This study |
| YLN073 | YEK014 + pEK17 + pLN14 + pEK18 | This study |
| YLN074 | YEK014 + pEK17 + pEK7 + pLN15 | This study |
| YLN075 | YEK014 + pEK36c + pLN14 + pEK18 | This study |
| YLN076 | YEK014 + pEK36c + pEK7 + pLN15 | This study |
| YLN077 | YEK014 + pEK17 + pLN14 + pLN15 | This study |
| YLN078 | YEK014 + pEK36c + pLN14 + pLN15 | This study |
| YLN079 | YEK014 + pEK7 + pEK17 + pEK18 | This study |
| YLN096 | W303α + pLN22 + pEK7 +pEK18 | This study |
| YLN097 | YEK014 + pLN22 + pEK7 + pEK18 | This study |
| YLN108 | YLN107 + pEK7 + pEK17 + pEK18 | This study |
| YLN109 | YLN107 + pEK36C + pEK7 + pEK18 | This study |
| YLN110 | YLN107 + pEK17 + pLN14 + pEK18 | This study |
| YLN111 | YLN107 + pEK17 + pEK7 + pLN15 | This study |
| YLN112 | YLN107 + pEK36c + pLN14 + pEK18 | This study |
| YLN113 | YLN107 + pEK36c + pEK7 + pLN15 | This study |
| YLN114 | YLN107 + pEK17 + pLN14 + pLN15 | This study |
| YLN115 | YLN107 + pEK36c + pLN14 + pLN15 | This study |
| YLN116 | YLN107 + pLN22 + pEK7 + pEK18 | This study |

Supplementary Table S1b. *Saccharomyces cerevisiae* strains used in this study.

| Name | Genotype or genetic modifications | Reference |
| --- | --- | --- |
| YLN118 | YLN117 + pEK7 + pEK17 + pEK18 | This study |
| YLN119 | YLN117 + pEK36C + pEK7 + pEK18 | This study |
| YLN120 | YLN117 + pEK17 + pLN14 + pEK18 | This study |
| YLN121 | YLN117 + pEK17 + pEK7 + pLN15 | This study |
| YLN122 | YLN117 + pEK36c + pLN14 + pEK18 | This study |
| YLN123 | YLN117 + pEK36c + pEK7 + pLN15 | This study |
| YLN124 | YLN117 + pEK17 + pLN14 + pLN15 | This study |
| YLN125 | YLN117 + pEK36c + pLN14 + pLN15 | This study |
| YLN129 | YMR024 + pEK7 + pEK17 + pEK18 | This study |
| YLN130 | YMR024 + pEK36C + pEK7 + pEK18 | This study |
| YLN131 | YMR024 + pEK17 + pLN14 + pEK18 | This study |
| YLN132 | YMR024 + pEK17 + pEK7 + pLN15 | This study |
| YLN133 | YMR024 + pEK36c + pLN14 + pEK18 | This study |
| YLN134 | YMR024 + pEK36c + pEK7 + pLN15 | This study |
| YLN135 | YMR024 + pEK17 + pLN14 + pLN15 | This study |
| YLN136 | YMR024 + pEK36c + pLN14 + pLN15 | This study |
| YLN137 | YMR024 + pLN22 + pEK7 + pEK18 | This study |
| YLN138 | YMR024 + pEK17 + pMS109 + pEK18 | This study |
| YLN139 | YLN127 + pEK17 + pEK7 + pEK18 | This study |
| YLN140 | YLN127 + pEK36C + pEK7 + pEK18 | This study |
| YLN141 | YLN127 + pEK17 + pLN14 + pEK18 | This study |
| YLN142 | YLN127 + pEK17 + pEK7 + pLN15 | This study |
| YLN143 | YLN127 + pEK36c + pLN14 + pEK18 | This study |
| YLN144 | YLN127 + pEK36c + pEK7 + pLN15 | This study |
| YLN145 | YLN127 + pEK17 + pLN14 + pLN15 | This study |
| YLN146 | YLN127 + pEK36c + pLN14 + pLN15 | This study |
| YLN147 | YLN127 + pLN22 + pEK7 + pEK18 | This study |
| YLN148 | YLN127 + pEK17 + pMS109 + pEK18 | This study |
| YLN149 | YLN126 + pEK17 + pEK7 + pEK18 | This study |
| YLN150 | YLN126 + pEK36C + pEK7 + pEK18 | This study |
| YLN151 | YLN126 + pEK17 + pLN14 + pEK18 | This study |
| YLN152 | YLN126 + pEK17 + pEK7 + pLN15 | This study |

Supplementary Table S1c. *Saccharomyces cerevisiae* strains used in this study.

| Name | Genotype or genetic modifications | Reference |
| --- | --- | --- |
| YLN153 | YLN126 + pEK36c + pLN14 + pEK18 | This study |
| YLN154 | YLN126 + pEK36c + pEK7 + pLN15 | This study |
| YLN155 | YLN126 + pEK17 + pLN14 + pLN15 | This study |
| YLN156 | YLN126 + pEK36c + pLN14 + pLN15 | This study |
| YLN157 | YLN126 + pLN22 + pEK7 + pEK18 | This study |
| YLN158 | YLN126 + pEK17 + pMS109 + pEK18 | This study |
| YLN159 | YLN128 + pEK17 + pEK7 + pEK18 | This study |
| YLN160 | YLN128 + pEK36C + pEK7 + pEK18 | This study |
| YLN161 | YLN128 + pEK17 + pLN14 + pEK18 | This study |
| YLN162 | YLN128 + pEK17 + pEK7 + pLN15 | This study |
| YLN163 | YLN128 + pEK36c + pLN14 + pEK18 | This study |
| YLN164 | YLN128 + pEK36c + pEK7 + pLN15 | This study |
| YLN165 | YLN128 + pEK17 + pLN14 + pLN15 | This study |
| YLN166 | YLN128 + pEK36c + pLN14 + pLN15 | This study |
| YLN167 | YLN128 + pEK17 + pMS109 + pEK18 | This study |

Supplementary Table S2. List of plasmids used in this study.

| Name | Description | Reference |
| --- | --- | --- |
| pEK18 | Control plasmid derived from pRS413-Tef (HIS3) without a promoter | [2] |
| pEK7 | Control plasmid derived from pRS415-Tef (LEU2) without a promoter | [2] |
| pEK17 | Control plasmid derived from pRS416-Tef (URA3) without a promoter | [2] |
| pLN15 | Derivative of pRS413-Tef (HIS3) expressing Yop1 | This study |
| pLN14 | Derivative of pRS415-Tef (LEU2) expressing Rtn2 | This study |
| pEK36C | Derivative of pRS416-Tef (URA3) expressing Rtn1 | This study |
| pLN22 | Derivative of pRS416-Tef (URA3) expressing Dgk1 | This study |
| pDEP017 | An integrative plasmid (TRP1 site) based on pRS304 plasmid with 4xUPRE in a crippled cyc1 and NdegY-GFP | [3] |
| pUG6 | KanMX marker casette flanked by loxP-sites | [4] |
| pUG74 | NatMX marker casette flanked by loxP-sites | [4] |
| pSH47 | Cre-expressing plasmid with URA3 marker | [4] |
| pMS109 | Constitutive HAC1^i^ expression plasmid with LEU2 marker | [5] |

Supplementary Table S3. Oligonucleotides used in this study.

| rtn1_fw | EK079 | AACTAGTGAGACC ATG TCCGCCTCAGCTC |
| --- | --- | --- |
| rtn1_rev | EK080 | AATTACATGACTCGAG TCAAGCGTTGTTTTTTTC |
| rtn2_fw | OLN5 | GGCGTACTAGTATGAATAGGAATACGACTACC |
| rtn2_rev | OLN6 | GGCAACTCGAGTTATGCGTGTTGCAATTTTTGC |
| yop1_fw | OLN7 | GGCTTGACTAGTATGTCCGAATATGCATCTAGTATTCAC |
| yop1_rev | OLN8 | GGCTACTCGAGTTAATGAACAGAAGCACCTGTAG |
| dgk1_fw | OAF98 | aaACTAGTATGGGGACCGAAGATGCCATTGC |
| dgk1_rev | OAF99 | aaTTATTTCTTGAATGTTTTGATTACTGC |
| tgl3_ko_fw | OLN9 | AGTAAGGGAATCATCTATTCATATATCACATCTTTGAGTT GCCGTTAAGCGTACGCTGCAGGTCGACAAC |
| tgl3_ko_rev | OLN10 | AATCGAGCTCTATCAATAAAAAAAATAAGACAGAAAAAA GTGGAAACGATAGCATAGGCCACTAGTGGATCTG |
| tgl4_ko_fw | OLN11 | GCGCTGTAATAATTATTGAAGGGAGTACAGGTATATGTAATAAAAGTCTGA CAGCTGAAGCTTCGTACGC |
| tgl4_ko_rev | OLN12 | CATAGATGAAAAAGAATATCTAGAGGATATATAAGCAAGCCCGTGTTTTCG CATAGGCCACTAGTGGATCTG |

Supplementary Table S4. Target enzymes and enzyme functions.

| Enzyme | Function | Reference |
| --- | --- | --- |
| Tgl3 | Tgl3 is a triacylglycerol lipase located on LDs. It is involved in the mobilization of neutral lipids from LDs by hydrolysis of TAGs, thereby releasing fatty acids. | [6] |
| Tgl4 | Tgl4 is a triacylglycerol lipase located on LDs. It is involved in the mobilization of neutral lipids from LDs by hydrolysis of TAGs, thereby releasing fatty acids. Tgl4 shows also steryl ester hydrolase and phospholipase A_2_ activities. | [6] |
| Dgk1 | Dgk1 is a transmembrane protein located at the ER membrane and the nuclear envelope. It is CTP-dependent and catalyzes the phosphorylation of DAG to PA. | [7] |

Supplementary Table S5. Expected outcomes of genetic modifications.

| Genotype/ genetic modifications | Expected outcome of modification | Reference |
| --- | --- | --- |
| WT | Control strain |  |
| WT Rtn1/ Rtn2/ Yop1 | We expected that overexpression of reticulons and/ or Yop1 would increase ER membrane curvature and raise the tubule to sheet ratio. We further anticipated that LDs would be smaller in size but more numerous. Moreover, we hypothesized that simultaneous overexpression of multiple membrane-curvature inducing proteins would amplify these effects on the associated structures. | [8] |
| WT Dgk1 | We anticipated the overexpression of Dgk1 to lead to an expansion of the ER membranes especially the perinuclear ER. | [7] |
| *Δopi1* | We expected the absence of the *OPI1* gene to lead to an expansion of the ER membranes. | [9] |
| *Δopi1* Rtn1/ Rtn2/ Yop1 | We expected an increase in tubule formation in comparison to the corresponding WT strain. We expected the strains overexpressing reticulons and Yop1 to increase LD formation and the formed LDs to be smaller in comparison to the control. | [8, 9] |
| *Δopi1* Dgk1 | We anticipated ER membrane expansion, and distinctly perinuclear ER expansion. | [10] |
| *Δtgl3Δtgl4* | We expected the absence of the lipases Tgl3 and Tgl4 result in larger LDs in comparison to the WT control. | [6] |
| *Δtgl3Δtgl4* Rtn1/ Rtn2/ Yop1 | We generally expected to observe larger LDs compared to the WT strain. Furthermore, we hypothesized that overexpression of membrane curvature inducing proteins would increase ER membrane curvature, resulting in a higher tubule to sheet ratio. We anticipated that overexpression of reticulons and/or Yop1 would produce a greater number of LDs, but of smaller size compared to the corresponding control. | [6, 8] |
| *Δtgl3Δtgl4* Dgk1 | We expected larger LDs in comparison to the WT and an expansion of the ER membranes especially of the perinuclear ER. | [6, 10] |
| *Δopi1Δtgl3Δtgl4* | We expected the LDs to be larger in *Δopi1Δtgl3Δtgl4* cells than in the WT control strain and that the ER membranes would be expanded in comparison to the WT cells. | [6, 9] |
| *Δopi1Δtgl3Δtgl4* Rtn1/ Rtn2/ Yop1 | In *Δopi1Δtgl3Δtgl4* cells overexpressing membrane curvature inducing proteins, we expected LDs to be larger than in the WT but smaller than in the corresponding control cells, accompanied by an increase in droplet number. We further anticipated expanded ER membranes and enhanced tubule formation relative to the WT strain. | [6, 8, 9] |
| *Δopi1Δtgl3Δtgl4* Dgk1 | We expected the LDs to be larger in comparison to the LDs in the WT cells and that the ER membranes expanded in comparison to the WT cells. We also expected distinct perinuclear ER expansion. | [6, 9, 10] |

Supplementary Table S6a. Strain specific cell and lipid droplet data. Data of strains YLN060-YLN097 is shown.

| Strain name | Genotype/ genetic modifications | Cell cross-sectional area average ± standard deviation (µm²) | Fold change cell cross-sectional area | Number of cells studied | LD cross-sectional area average ± standard deviation (µm²) | Fold change LD cross-sectional area | Number of LDs studied | Lipid content average ± standard deviation (mg/ mg) | Fold change lipid content |
| --- | --- | --- | --- | --- | --- | --- | --- | --- | --- |
| YLN060 | WT | 27.41±7.57 | 1.00 | 100 | 0.20±0.08 | 1.00 | 135 | 0.14±0.02 | 1.00 |
| YLN061 | WT Rtn1 | 27.21±10.68 | 0.99 | 90 | 0.15±0.05* | 0.72 | 71 | 0.23±0.06 | 1.69 |
| YLN062 | WT Rtn2 | 32.02±12.17 | 1.17 | 90 | 0.16±0.07 | 0.80 | 86 | 0.12±0.01 | 0.89 |
| YLN063 | WT Yop1 | 27.70±10.05 | 1.01 | 123 | 0.17±0.05 | 0.84 | 81 | 0.18±0.05 | 1.30 |
| YLN064 | WT Rtn1Rtn2 | 29.88±11.66 | 1.09 | 83 | 0.15±0.05* | 0.74 | 53 | 0.22±0.09 | 1.62 |
| YLN065 | WT Rtn1Yop1 | 28.05±10.13 | 1.02 | 100 | 0.15±0.04 | 0.75 | 65 | 0.25±0.11 | 1.81 |
| YLN066 | WT Rtn2Yop1 | 23.77±10.25 | 0.87 | 138 | 0.14±0.07* | 0.69 | 102 | 0.16±0.03 | 1.17 |
| YLN067 | WT Rtn1Rtn2Yop1 | 22.70±7.54 | 0.83 | 108 | 0.12±0.05* | 0.60 | 105 | 0.19±0.03 | 1.39 |
| YLN096 | WT Dgk1 | 28.72±9.06 | 1.05 | 114 | 0.15±0.05* | 0.76 | 87 | 0.12±0.04 | 0.89 |
| YLN079 | *Δopi1* | 34.85±7.85 | 1.00 | 75 | 0.19±0.10 | 1.00 | 38 | 0.11±0.01 | 1.00 |
| YLN072 | *Δopi1* Rtn1 | 36.82±8.31 | 1.06 | 93 | 0.20±0.06 | 1.05 | 48 | 0.11±0.02 | 1.03 |
| YLN073 | *Δopi1* Rtn2 | 35.31±9.23 | 1.01 | 99 | 0.17±0.05 | 0.90 | 52 | 0.13±0.03 | 1.21 |
| YLN074 | *Δopi1* Yop1 | 36.07±9.43 | 1.03 | 80 | 0.14±0.05 | 0.77 | 46 | 0.13±0.02 | 1.15 |
| YLN075 | *Δopi1* Rtn1Rtn2 | 44.00±12.24* | 1.26 | 85 | 0.13±0.04 | 0.71 | 45 | 0.09±0.02 | 0.85 |
| YLN076 | *Δopi1* Rtn1Yop1 | 36.92±9.96 | 1.06 | 130 | 0.11±0.04* | 0.62 | 68 | 0.12±0.04 | 1.13 |
| YLN077 | *Δopi1* Rtn2Yop1 | 45.82±8.94* | 1.31 | 59 | 0.10±0.03* | 0.50 | 49 | 0.13±0.02 | 1.15 |
| YLN078 | *Δopi1* Rtn1Rtn2Yop1 | 34.79±7.14 | 1.00 | 60 | 0.10±0.03* | 0.55 | 118 | 0.13±0.05 | 1.23 |
| YLN097 | *Δopi1* Dgk1 | 40.23±11.09 | 1.15 | 86 | 0.14±0.04 | 0.75 | 105 | 0.10±0.04 | 0.88 |

Supplementary Table S6b. Strain specific cell and lipid droplet data. Data of strains YLN108-YLN125 is shown.

| Strain name | Genotype/ genetic modifications | Cell cross-sectional area average ± standard deviation (µm²) | Fold change cell cross-sectional area | Number of cells studied | LD cross-sectional area average ± standard deviation (µm²) | Fold change LD cross-sectional area | Number of LDs studied | Lipid content average ± standard deviation (mg/ mg) | Fold change lipid content |
| --- | --- | --- | --- | --- | --- | --- | --- | --- | --- |
| YLN108 | *Δtgl3Δtgl4* | 29.10±10.00 | 1.00 | 94 | 0.30±0.10 | 1.00 | 184 | 0.16±0.09 | 1.00 |
| YLN109 | *Δtgl3Δtgl4* Rtn1 | 31.92±9.95 | 1.10 | 101 | 0.33±0.15 | 1.09 | 192 | 0.14±0.02 | 0.85 |
| YLN110 | *Δtgl3Δtgl4* Rtn2 | 28.22±11.58 | 0.97 | 83 | 0.33±0.16 | 1.10 | 186 | 0.12±0.03 | 0.74 |
| YLN111 | *Δtgl3Δtgl4* Yop1 | 26.66±10.06 | 0.92 | 117 | 0.31±0.12 | 1.03 | 251 | 0.17±0.06 | 1.01 |
| YLN112 | *Δtgl3Δtgl4* Rtn1Rtn2 | 24.85±8.06 | 0.85 | 72 | 0.28±0.17 | 0.91 | 50 | 0.16±0.05 | 0.95 |
| YLN113 | *Δtgl3Δtgl4* Rtn1Yop1 | 24.61±7.07 | 0.85 | 81 | 0.23±0.08* | 0.77 | 152 | 0.14±0.02 | 0.86 |
| YLN114 | *Δtgl3Δtgl4* Rtn2Yop1 | 25.53±10.00 | 0.88 | 65 | 0.20±0.07* | 0.67 | 268 | 0.16±0.03 | 0.96 |
| YLN115 | *Δtgl3Δtgl4* Rtn1Rtn2Yop1 | 25.75±10.43 | 0.88 | 122 | 0.20±0.07* | 0.67 | 174 | 0.13±0.01 | 0.79 |
| YLN116 | *Δtgl3Δtgl4* Dgk1 | 32.84±15.07 | 1.13 | 98 | 0.31±0.13 | 1.01 | 109 | 0.19±0.08 | 1.17 |
| YLN118 | *Δopi1Δtgl3Δtgl4* | 36.01±13.75 | 1.00 | 99 | 0.30±0.10 | 1.00 | 94 | 0.099±0.03 | 1.00 |
| YLN119 | *Δopi1Δtgl3Δtgl4* Rtn1 | 48.27±21.96* | 1.34 | 153 | 0.27±0.07 | 0.90 | 42 | 0.13±0.04 | 1.29 |
| YLN120 | *Δopi1Δtgl3Δtgl4* Rtn2 | 49.84±17.07* | 1.38 | 84 | 0.21±0.11* | 0.70 | 52 | 0.14±0.08 | 1.40 |
| YLN121 | *Δopi1Δtgl3Δtgl4* Yop1 | 58.37±23.62* | 1.62 | 102 | 0.17±0.05* | 0.56 | 108 | 0.15±0.08 | 1.54 |
| YLN122 | *Δopi1Δtgl3Δtgl4* Rtn1Rtn2 | 44.68±22.40* | 1.24 | 100 | 0.19±0.11* | 0.64 | 114 | 0.18±0.14 | 1.82 |
| YLN123 | *Δopi1Δtgl3Δtgl4* Rtn1Yop1 | 44.17±16.86* | 1.23 | 77 | 0.16±0.09* | 0.54 | 65 | 0.14±0.05 | 1.46 |
| YLN124 | *Δopi1Δtgl3Δtgl4* Rtn2Yop1 | 53.26±21.08* | 1.48 | 72 | 0.15±0.04* | 0.49 | 78 | 0.12±0.06 | 1.17 |
| YLN125 | *Δopi1Δtgl3Δtgl4* Rtn1Rtn2Yop1 | 48.68±20.51* | 1.35 | 82 | 0.19±0.14* | 0.63 | 44 | 0.11±0.02 | 1.15 |

Supplementary Table S7a. Fatty acid composition after 48 h of nitrogen starvation of samples produced by strains YLN060-YLN097

| **Strain**  **name** | **C12:0** | **C14:0** | **C14:1** | **C16:0** | **C16:1** | **C18:0** | **C18:1** | **UFA** | **SFA** |  | **C16:1/C16:0** |
| --- | --- | --- | --- | --- | --- | --- | --- | --- | --- | --- | --- |
| **YLN060** | 1.041±0.438 | 2.319±1.073 | 0.692±0.309 | 19.569±4.107 | 28.294±3.211 | 3.786±0.699 | 44.298±4.939 | 73.285 | 26.715 | ±6.017 | 1.446 |
| **YLN061** | 1.188±0.428 | 2.795±1.147 | 0.963±0.453 | 18.467±3.502 | 24.734±2.261 | 3.135±0.728 | 48.718±5.363 | 74.415 | 25.585 | ±5.445 | 1.339 |
| **YLN062** | 1.028±0.270 | 2.195±0.818 | 0.689±0.262 | 19.693±3.004 | 29.130±2.375 | 4.195±0.639 | 43.071±3.475 | 72.889 | 27.111 | ±4.506 | 1.479 |
| **YLN063** | 1.124±0.367 | 2.276±0.791 | 0.693±0.262 | 19.129±2.443 | 28.015±2.634 | 3.816±0.385 | 44.946±3.227 | 73.655 | 26.345 | ±3.784 | 1.465 |
| **YLN064** | 1.046±0.219 | 2.320±0.378 | 0.772±0.244 | 19.220±2.563 | 26.543±2.768 | 3.583±0.694 | 46.515±5.218 | 73.830 | 26.170 | ±3.271 | 1.381 |
| **YLN065** | 1.247±0.340 | 2.542±0.742 | 0.885±0.344 | 18.628±1.751 | 25.972±3.857 | 3.567±0.970 | 47.159±5.209 | 74.016 | 25.984 | ±2.913 | 1.394 |
| **YLN066** | 1.165±0.208 | 2.462±0.384 | 0.791±0.199 | 19.588±2.636 | 25.992±2.482 | 3.597±0.796 | 46.405±5.692 | 73.187 | 26.813 | ±3.626 | 1.327 |
| **YLN067** | 1.043±0.230 | 2.417±0.661 | 0.853±0.331 | 19.203±1.840 | 27.461±1.987 | 3.681±0.562 | 45.342±1.671 | 73.656 | 26.344 | ±2.749 | 1.430 |
| **YLN096** | 1.746±0.816 | 3.348±0.606 | 1.037±0.257 | 21.779±0.749 | 24.179±1.384 | 3.573±0.139 | 44.337±2.505 | 69.553 | 30.447 | ±1.673 | 1.110 |
| **YLN079** | 1.751±0.216 | 3.504±0.698 | 0.698±0.273 | 22.485±1.014 | 23.016±2.130 | 3.581±0.372 | 44.631±0.756 | 68.679 | 31.321 | ±1.338 | 1.024 |
| **YLN072** | 1.710±0.344 | 3.323±0.762 | 1.005±0.264 | 20.971±2.272 | 22.673±2.173 | 3.331±0.517 | 46.986±4.615 | 70.664 | 29.336 | ±2.879 | 1.081 |
| **YLN073** | 1.789±0.348 | 3.515±0.879 | 1.073±0.293 | 21.295±1.749 | 22.747±1.379 | 3.219±0.376 | 46.362±2.649 | 70.182 | 29.818 | ±2.686 | 1.068 |
| **YLN074** | 1.500±0.238 | 3.082±0.502 | 0.879±0.182 | 21.979±1.506 | 24.620±1.832 | 3.844±0.300 | 44.096±2.847 | 69.595 | 30.405 | ±1.927 | 1.120 |
| **YLN075** | 1.512±0.247 | 3.070±0.727 | 0.964±0.306 | 21.169±1.943 | 24.097±0.809 | 3.521±0.352 | 45.666±3.151 | 70.727 | 29.273 | ±3.045 | 1.138 |
| **YLN076** | 1.503±0.331 | 3.192±0.964 | 0.989±0.355 | 21.716±1.551 | 24.136±1.679 | 3.759±0.280 | 44.706±2.561 | 69.831 | 30.169 | ±2.679 | 1.111 |
| **YLN077** | 1.427±0.220 | 3.078±0.834 | 0.922±0.267 | 20.945±1.329 | 23.727±1.955 | 3.523±0.598 | 46.378±1.522 | 71.027 | 28.973 | ±1.560 | 1.133 |
| **YLN078** | 1.395±0.274 | 2.984±0.563 | 0.925±0.220 | 21.302±1.893 | 23.552±0.485 | 3.543±0.508 | 46.300±2.542 | 70.777 | 29.223 | ±2.406 | 1.106 |
| **YLN097** | 1.729±0.274 | 3.435±0.686 | 1.010±0.256 | 21.932±1.759 | 22.795±2.171 | 3.246±0.527 | 45.852±4.202 | 69.657 | 30.343 | ±2.782 | 1.039 |

Supplementary Table S7b. Fatty acid composition after 48 h of nitrogen starvation of samples produced by strains YLN108-YLN125

| **Strain name** | **C12:0** | **C14:0** | **C14:1** | **C16:0** | **C16:1** | **C18:0** | **C18:1** | **UFA** | **SFA** |  | **C16:1/C16:0** |
| --- | --- | --- | --- | --- | --- | --- | --- | --- | --- | --- | --- |
| **YLN108** | 0.910±0.217 | 2.484±0.310 | 0.850±0.149 | 18.446±1.179 | 25.664±3.070 | 3.822±1.205 | 47.822±4.954 | 74.337 | 25.663 | ±2.036 | 1.391 |
| **YLN109** | 1.141±0.277 | 2.884±0.382 | 0.993±0.102 | 17.703±2.139 | 24.217±3.119 | 3.152±0.778 | 49.909±5.767 | 75.119 | 24.881 | ±2.959 | 1.368 |
| **YLN110** | 1.002±0.153 | 2.662±0.311 | 0.940±0.097 | 18.281±1.360 | 25.392±1.833 | 3.577±0.755 | 48.146±3.679 | 74.478 | 25.522 | ±2.128 | 1.389 |
| **YLN111** | 0.924±0.250 | 2.457±0.256 | 0.825±0.076 | 18.370±1.188 | 26.185±2.365 | 3.490±0.788 | 47.750±3.709 | 74.759 | 25.241 | ±2.007 | 1.425 |
| **YLN112** | 1.090±0.158 | 2.743±0.361 | 0.999±0.207 | 17.744±0.994 | 24.660±2.222 | 3.153±0.640 | 49.611±3.020 | 75.270 | 24.730 | ±1.490 | 1.390 |
| **YLN113** | 1.059±0.166 | 2.702±0.549 | 1.008±0.200 | 16.992±1.347 | 25.242±2.305 | 3.235±0.598 | 49.762±3.586 | 76.012 | 23.988 | ±2.119 | 1.486 |
| **YLN114** | 1.195±0.347 | 2.844±0.433 | 1.019±0.217 | 16.704±1.642 | 23.789±3.546 | 3.027±0.754 | 51.423±5.284 | 76.230 | 23.770 | ±2.478 | 1.424 |
| **YLN115** | 0.898±0.186 | 2.436±0.267 | 0.931±0.072 | 17.280±1.072 | 26.059±2.713 | 3.470±0.488 | 48.925±3.415 | 75.915 | 24.085 | ±1.339 | 1.508 |
| **YLN116** | 1.128±0.328 | 2.676±0.260 | 0.921±0.135 | 17.827±1.883 | 23.383±3.746 | 2.861±0.658 | 51.203±5.539 | 75.508 | 24.492 | ±2.309 | 1.312 |
| **YLN118** | 0.685±0.062 | 2.031±0.255 | 0.469±0.065 | 24.648±0.735 | 23.193±3.170 | 4.434±1.272 | 44.826±3.965 | 68.372 | 31.628 | ±1.575 | 0.941 |
| **YLN119** | 0.690±0.129 | 2.170±0.136 | 0.539±0.038 | 23.485±0.459 | 22.999±1.042 | 4.347±0.389 | 45.942±1.562 | 69.480 | 30.520 | ±0.720 | 0.979 |
| **YLN120** | 0.633±0.253 | 2.414±0.225 | 0.504±0.083 | 24.364±0.363 | 21.552±2.134 | 3.723±1.046 | 46.810±2.900 | 68.866 | 31.134 | ±0.909 | 0.885 |
| **YLN121** | 0.821±0.181 | 2.620±0.174 | 0.697±0.043 | 23.221±1.130 | 21.667±2.705 | 3.530±0.821 | 47.444±3.899 | 69.808 | 30.192 | ±1.520 | 0.933 |
| **YLN122** | 0.719±0.155 | 2.361±0.080 | 0.568±0.019 | 23.038±1.294 | 22.478±1.441 | 4.081±0.634 | 46.755±3.274 | 69.801 | 30.199 | ±1.841 | 0.976 |
| **YLN123** | 0.678±0.427 | 2.290±0.369 | 0.601±0.146 | 21.769±1.358 | 22.603±4.220 | 4.252±1.570 | 47.807±5.748 | 71.012 | 28.988 | ±1.805 | 1.038 |
| **YLN124** | 0.943±0.153 | 2.485±0.340 | 0.598±0.127 | 22.960±2.077 | 24.036±2.286 | 4.242±0.293 | 44.737±2.132 | 69.371 | 30.629 | ±2.441 | 1.047 |
| **YLN125** | 0.771±0.121 | 2.181±0.206 | 0.446±0.261 | 21.220±1.673 | 24.351±3.153 | 3.705±0.397 | 47.327±3.193 | 72.124 | 27.876 | ±1.898 | 1.148 |

Supplementary Table S8a. Strain specific maximum GFP levels during time course analysis of the UPR with comparisons to the corresponding control of the strains YLN129-YLN138 carrying the GFP based UPR responsive transcriptional reporter.

| Strain name | Genotype/ genetic modifications | Induction | GFP level Ymax (RFL) | GFP level  Y_max_ – Y_0_ (RFL) | Fold change GFP level |
| --- | --- | --- | --- | --- | --- |
| YLN129 | WT | DTT | 278.2 | 165.2 | 1.0 |
| YLN130 | WT Rtn1 | DTT | 307.5 | 148.1 | 0.9 |
| YLN131 | WT Rtn2 | DTT | 387.5 | 320.0 | 1.9 |
| YLN132 | WT Yop1 | DTT | 478.3 | 351.2 | 2.1 |
| YLN133 | WT Rtn1Rtn2 | DTT | 482.8 | 372.6 | 2.3 |
| YLN134 | WT Rtn1Yop1 | DTT | 502.4 | 271.8 | 1.6 |
| YLN135 | WT Rtn2Yop1 | DTT | 499.7 | 336.2 | 2.0 |
| YLN136 | WT Rtn1Rtn2Yop1 | DTT | 732.0 | 528.7 | 3.2 |
| YLN137 | WT Dgk1 | DTT | 254.4 | 129.6 | 0.8 |
| YLN138 | WT Hac1 | DTT | 990.5 | 736.5 | 4.6 |
| YLN129 | WT | Tween-80 | 327.1 | 175.3 | 1.0 |
| YLN130 | WT Rtn1 | Tween-80 | 316.5 | 119.7 | 0.7 |
| YLN131 | WT Rtn2 | Tween-80 | 348.2 | 245.3 | 1.4 |
| YLN132 | WT Yop1 | Tween-80 | 431.8 | 278.5 | 1.6 |
| YLN133 | WT Rtn1Rtn2 | Tween-80 | 413.5 | 261.1 | 1.5 |
| YLN134 | WT Rtn1Yop1 | Tween-80 | 462.4 | 186.8 | 1.1 |
| YLN135 | WT Rtn2Yop1 | Tween-80 | 462.9 | 254.5 | 1.5 |
| YLN136 | WT Rtn1Rtn2Yop1 | Tween-80 | 583.1 | 308.9 | 1.8 |
| YLN137 | WT Dgk1 | Tween-80 | 448.1 | 252.8 | 1.4 |
| YLN138 | WT Hac1 | Tween-80 | 1156.0 | 907.0 | 5.2 |
| YLN129 | WT | - | 233.7 | 135.2 | 1.0 |
| YLN130 | WT Rtn1 | - | 212.3 | 43.2 | 0.3 |
| YLN131 | WT Rtn2 | - | 293.4 | 233.27 | 1.7 |
| YLN132 | WT Yop1 | - | 321.5 | 190.5 | 1.4 |
| YLN133 | WT Rtn1Rtn2 | - | 324.1 | 207.9 | 1.5 |
| YLN134 | WT Rtn1Yop1 | - | 367.0 | 124.5 | 0.9 |
| YLN135 | WT Rtn2Yop1 | - | 381.7 | 196.5 | 1.5 |
| YLN136 | WT Rtn1Rtn2Yop1 | - | 538.4 | 312.6 | 2.3 |
| YLN137 | WT Dgk1 | - | 214.8 | 78.4 | 0.6 |
| YLN138 | WT Hac1 | - | 996.9 | 826.2 | 6.1 |

Supplementary Table S8b. Strain specific maximum GFP levels during time course analysis of the UPR with comparisons to the corresponding control of the strains YLN149-YLN158 carrying the GFP based UPR responsive transcriptional reporter.

| Strain name | Genotype/ genetic modifications | Induction | GFP level Ymax (RFL) | GFP level Y_max_ – Y_0_ (RFL) | Fold change GFP level |
| --- | --- | --- | --- | --- | --- |
| YLN149 | *Δopi1* | DTT | 329.7 | 271.9 | 1.0 |
| YLN150 | *Δopi1* Rtn1 | DTT | 393.9 | 323.1 | 1.2 |
| YLN151 | *Δopi1* Rtn2 | DTT | 417.0 | 347.1 | 1.3 |
| YLN152 | *Δopi1* Yop1 | DTT | 387.4 | 330.4 | 1.2 |
| YLN153 | *Δopi1* Rtn1Rtn2 | DTT | 430.5 | 368.8 | 1.4 |
| YLN154 | *Δopi1* Rtn1Yop1 | DTT | 454.7 | 375.7 | 1.4 |
| YLN155 | *Δopi1* Rtn2Yop1 | DTT | 453.2 | 383.0 | 1.4 |
| YLN156 | Δopi1 Rtn1Rtn2Yop1 | DTT | 384.0 | 344.5 | 1.3 |
| YLN157 | *Δopi1* Dgk1 | DTT | 367.8 | 327.8 | 1.2 |
| YLN158 | *Δopi1* Hac1 | DTT | 839.2 | 668.5 | 2.5 |
| YLN149 | *Δopi1* | Tween-80 | 324.8 | 267.4 | 1.0 |
| YLN150 | *Δopi1* Rtn1 | Tween-80 | 394.8 | 317.7 | 1.2 |
| YLN151 | *Δopi1* Rtn2 | Tween-80 | 409.2 | 322.5 | 1.2 |
| YLN152 | *Δopi1* Yop1 | Tween-80 | 368.7 | 304.6 | 1.1 |
| YLN153 | *Δopi1* Rtn1Rtn2 | Tween-80 | 433.5 | 349.6 | 1.3 |
| YLN154 | *Δopi1* Rtn1Yop1 | Tween-80 | 438.5 | 350.6 | 1.3 |
| YLN155 | *Δopi1* Rtn2Yop1 | Tween-80 | 441.0 | 360.6 | 1.4 |
| YLN156 | *Δopi1* Rtn1Rtn2Yop1 | Tween-80 | 387.8 | 326.8 | 1.2 |
| YLN157 | *Δopi1* Dgk1 | Tween-80 | 345.5 | 307.3 | 1.2 |
| YLN158 | *Δopi1* Hac1 | Tween-80 | 980.9 | 769.5 | 2.9 |
| YLN149 | *Δopi1* | - | 273.7 | 238.9 | 1.0 |
| YLN150 | *Δopi1* Rtn1 | - | 318.5 | 274.5 | 1.2 |
| YLN151 | *Δopi1* Rtn2 | - | 339.4 | 296.4 | 1.2 |
| YLN152 | *Δopi1* Yop1 | - | 307.8 | 274.8 | 1.2 |
| YLN153 | *Δopi1* Rtn1Rtn2 | - | 348.6 | 302.0 | 1.3 |
| YLN154 | *Δopi1* Rtn1Yop1 | - | 371.7 | 324.0 | 1.4 |
| YLN155 | *Δopi1* Rtn2Yop1 | - | 369.0 | 333.8 | 1.4 |
| YLN156 | Δopi1 Rtn1Rtn2Yop1 | - | 318.0 | 297.2 | 1.2 |
| YLN157 | *Δopi1* Dgk1 | - | 269.0 | 263.8 | 1.1 |
| YLN158 | *Δopi1* Hac1 | - | 892.8 | 741.4 | 3.1 |

Supplementary Table S8c. Strain specific maximum GFP levels during time course analysis of the UPR with comparisons to the corresponding control of the strains YLN139-YLN148 carrying the GFP based UPR responsive transcriptional reporter.

| Strain name | Genotype/ genetic modifications | Induction | GFP level Ymax (RFL) | GFP level Y_max_ – Y_0_ (RFL) | Fold change GFP level |
| --- | --- | --- | --- | --- | --- |
| YLN139 | *Δtgl3Δtgl4* | DTT | 459.8 | 425.0 | 1.0 |
| YLN140 | *Δtgl3Δtgl4* Rtn1 | DTT | 588.9 | 406.8 | 1.0 |
| YLN141 | *Δtgl3Δtgl4* Rtn2 | DTT | 505.4 | 443.7 | 1.0 |
| YLN142 | *Δtgl3Δtgl4* Yop1 | DTT | 390.5 | 255.6 | 0.6 |
| YLN143 | *Δtgl3Δtgl4* Rtn1Rtn2 | DTT | 770.3 | 676.4 | 1.6 |
| YLN144 | *Δtgl3Δtgl4* Rtn1Yop1 | DTT | 508.8 | 416.2 | 1.0 |
| YLN145 | *Δtgl3Δtgl4* Rtn2Yop1 | DTT | 625.8 | 563.8 | 1.3 |
| YLN146 | *Δtgl3Δtgl4* Rtn1Rtn2Yop1 | DTT | 646.6 | 474.4 | 1.1 |
| YLN147 | *Δtgl3Δtgl4* Dgk1 | DTT | 668.2 | 571.9 | 1.4 |
| YLN148 | *Δtgl3Δtgl4* Hac1 | DTT | 733.7 | 599.1 | 1.4 |
| YLN139 | *Δtgl3Δtgl4* | Tween-80 | 270.4 | 182.0 | 1.0 |
| YLN140 | *Δtgl3Δtgl4* Rtn1 | Tween-80 | 408.2 | 129.2 | 0.7 |
| YLN141 | *Δtgl3Δtgl4* Rtn2 | Tween-80 | 302.4 | 128.9 | 0.7 |
| YLN142 | *Δtgl3Δtgl4* Yop1 | Tween-80 | 259.8 | 30.9 | 0.2 |
| YLN143 | *Δtgl3Δtgl4* Rtn1Rtn2 | Tween-80 | 364.5 | 142.2 | 0.8 |
| YLN144 | *Δtgl3Δtgl4* Rtn1Yop1 | Tween-80 | 308.1 | 99.6 | 0.6 |
| YLN145 | *Δtgl3Δtgl4* Rtn2Yop1 | Tween-80 | 365.7 | 212.1 | 1.2 |
| YLN146 | *Δtgl3Δtgl4* Rtn1Rtn2Yop1 | Tween-80 | 442.5 | 132.1 | 0.7 |
| YLN147 | *Δtgl3Δtgl4* Dgk1 | Tween-80 | 382.3 | 216.9 | 1.2 |
| YLN148 | *Δtgl3Δtgl4* Hac1 | Tween-80 | 688.8 | 502.2 | 2.8 |
| YLN139 | *Δtgl3Δtgl4* | - | 200.7 | 151.9 | 1.0 |
| YLN140 | *Δtgl3Δtgl4* Rtn1 | - | 319.3 | 108.0 | 0.7 |
| YLN141 | *Δtgl3Δtgl4* Rtn2 | - | 261.3 | 131.9 | 0.9 |
| YLN142 | *Δtgl3Δtgl4* Yop1 | - | 194.7 | 44.9 | 0.3 |
| YLN143 | *Δtgl3Δtgl4* Rtn1Rtn2 | - | 312.5 | 150.4 | 1.0 |
| YLN144 | *Δtgl3Δtgl4* Rtn1Yop1 | - | 235.3 | 92.5 | 0.6 |
| YLN145 | *Δtgl3Δtgl4* Rtn2Yop1 | - | 303.0 | 200.1 | 1.3 |
| YLN146 | *Δtgl3Δtgl4* Rtn1Rtn2Yop1 | - | 334.8 | 117.9 | 0.8 |
| YLN147 | *Δtgl3Δtgl4* Dgk1 | - | 297.6 | 202.5 | 1.3 |
| YLN148 | *Δtgl3Δtgl4* Hac1 | - | 626.4 | 492.8 | 3.2 |

Supplementary Table S8d. Strain specific maximum GFP levels during time course analysis of the UPR with comparisons to the corresponding control of the strains YLN159-YLN167 carrying the GFP based UPR responsive transcriptional reporter.

| Strain name | Genotype/ genetic modifications | Induction | GFP level Ymax (RFL) | GFP level Y_max_ – Y_0_ (RFL) | Fold change GFP level |
| --- | --- | --- | --- | --- | --- |
| YLN159 | *Δopi1Δtgl3Δtgl4* | DTT | 578.3 | 519.0 | 1.0 |
| YLN160 | *Δopi1Δtgl3Δtgl4* Rtn1 | DTT | 726.3 | 599.6 | 1.2 |
| YLN161 | *Δopi1Δtgl3Δtgl4* Rtn2 | DTT | 608.2 | 519.7 | 1.0 |
| YLN162 | *Δopi1Δtgl3Δtgl4* Yop1 | DTT | 724.4 | 584.4 | 1.1 |
| YLN163 | *Δopi1Δtgl3Δtgl4* Rtn1Rtn2 | DTT | 804.9 | 629.3 | 1.2 |
| YLN164 | *Δopi1Δtgl3Δtgl4* Rtn1Yop1 | DTT | 702.1 | 569.5 | 1.1 |
| YLN165 | *Δopi1Δtgl3Δtgl4* Rtn2Yop1 | DTT | 900.5 | 686.7 | 1.3 |
| YLN166 | *Δopi1Δtgl3Δtgl4* Rtn1Rtn2Yop1 | DTT | 739.0 | 529.2 | 1.0 |
| YLN167 | *Δopi1Δtgl3Δtgl4* Hac1 | DTT | 748.8 | 594.0 | 1.1 |
| YLN159 | *Δopi1Δtgl3Δtgl4* | Tween-80 | 533.8 | 442.4 | 1.0 |
| YLN160 | *Δopi1Δtgl3Δtgl4* Rtn1 | Tween-80 | 604.8 | 411.7 | 0.9 |
| YLN161 | *Δopi1Δtgl3Δtgl4* Rtn2 | Tween-80 | 463.9 | 345.9 | 0.8 |
| YLN162 | *Δopi1Δtgl3Δtgl4* Yop1 | Tween-80 | 641.0 | 430.6 | 1.0 |
| YLN163 | *Δopi1Δtgl3Δtgl4* Rtn1Rtn2 | Tween-80 | 651.6 | 397.5 | 0.9 |
| YLN164 | *Δopi1Δtgl3Δtgl4* Rtn1Yop1 | Tween-80 | 563.7 | 368.7 | 0.8 |
| YLN165 | *Δopi1Δtgl3Δtgl4* Rtn2Yop1 | Tween-80 | 677.1 | 393.0 | 0.9 |
| YLN166 | *Δopi1Δtgl3Δtgl4* Rtn1Rtn2Yop1 | Tween-80 | 626.2 | 351.6 | 0.8 |
| YLN167 | *Δopi1Δtgl3Δtgl4* Hac1 | Tween-80 | 651.7 | 475.2 | 1.1 |
| YLN159 | *Δopi1Δtgl3Δtgl4* | - | 452.1 | 397.6 | 1.0 |
| YLN160 | *Δopi1Δtgl3Δtgl4* Rtn1 | - | 546.3 | 404.0 | 1.0 |
| YLN161 | *Δopi1Δtgl3Δtgl4* Rtn2 | - | 427.8 | 347.1 | 0.9 |
| YLN162 | *Δopi1Δtgl3Δtgl4* Yop1 | - | 611.7 | 451.7 | 1.1 |
| YLN163 | *Δopi1Δtgl3Δtgl4* Rtn1Rtn2 | - | 585.1 | 373.9 | 0.9 |
| YLN164 | *Δopi1Δtgl3Δtgl4* Rtn1Yop1 | - | 500.1 | 347.6 | 0.9 |
| YLN165 | *Δopi1Δtgl3Δtgl4* Rtn2Yop1 | - | 615.3 | 379.2 | 1.0 |
| YLN166 | *Δopi1Δtgl3Δtgl4* Rtn1Rtn2Yop1 | - | 605.0 | 347.3 | 0.9 |
| YLN167 | *Δopi1Δtgl3Δtgl4* Hac1 | - | 593.2 | 455.9 | 1.2 |


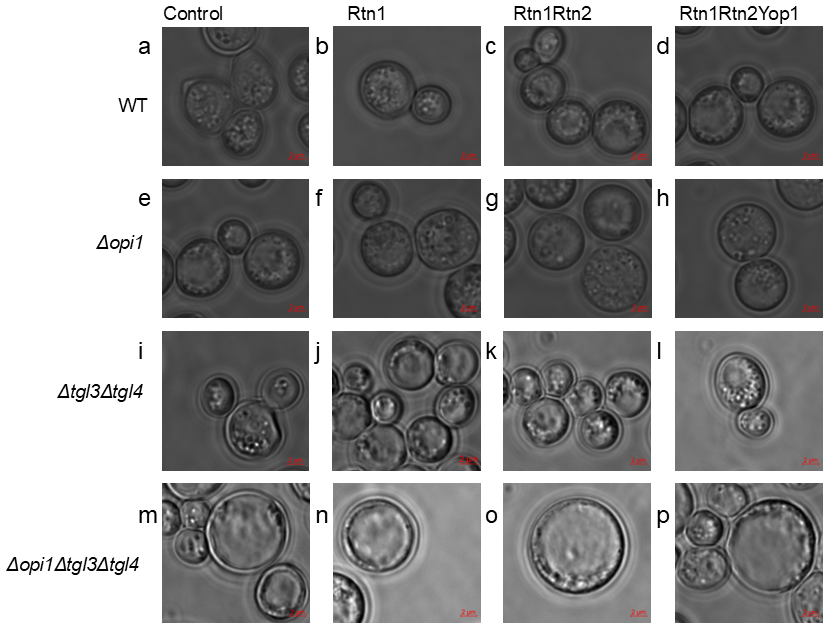


Supplementary Figure S1. *S. cerevisiae* strains overexpressing membrane curvature inducing proteins and their corresponding controls. Panels a – d present selected strains in the wt background: a) WT control; b) Rtn1; c) Rtn1Rtn2; d) Rtn1Rtn2Yop1. Panels e – h present selected strains in the *Δopi1* background: a) *Δopi1* control; b) *Δopi1* Rtn1; c) *Δopi1* Rtn1Rtn2; d) *Δopi1* Rtn1Rtn2Yop1. Panels i – l present selected strains in the *Δtgl3Δtgl4* background: i) *Δtgl3Δtgl4* control; j) *Δtgl3Δtgl4* Rtn1; k) *Δtgl3Δtgl4* Rtn1Rtn2; l) *Δtgl3Δtgl4* Rtn1Rtn2Yop1. Panels m – p present selected strains in the *Δopi1Δtgl3Δtgl4* background: m) *Δopi1Δtgl3Δtgl4* control; n) *Δopi1Δtgl3Δtgl4* Rtn1; o) *Δopi1Δtgl3Δtgl4* Rtn1Rtn2; p) *Δopi1Δtgl3Δtgl4* Rtn1Rtn2Yop1. Scale bar: 2 µm.


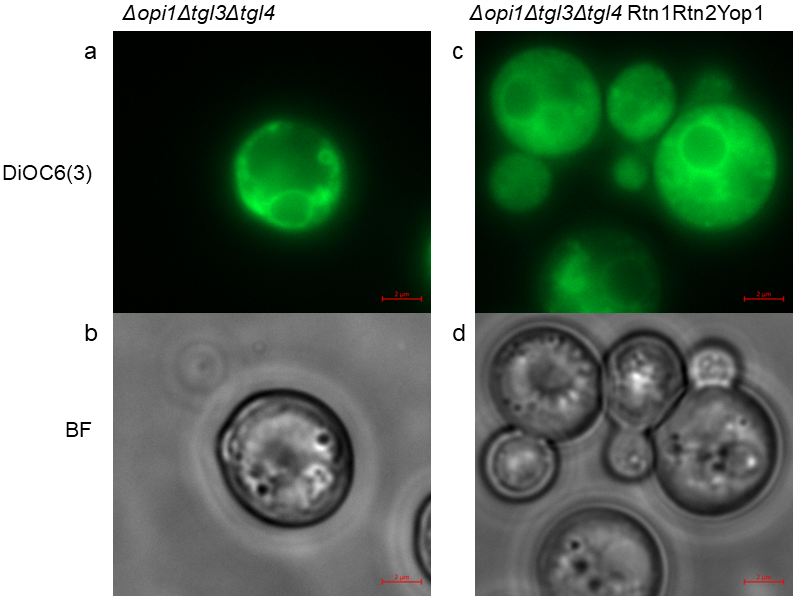
Supplementary figure S2. DiOC6(3) stained structures in the *Δopi1Δtgl3Δtgl4* strain background cells. Panels a - b present the control strain in a) fluorescence mode b) brightfield mode. Panels c – d present the *Δopi1Δtgl3Δtgl4* Rtn1Rtn2Yop1 strain in c) fluorescence mode d) brightfield mode. Scale bar: 2 µm.


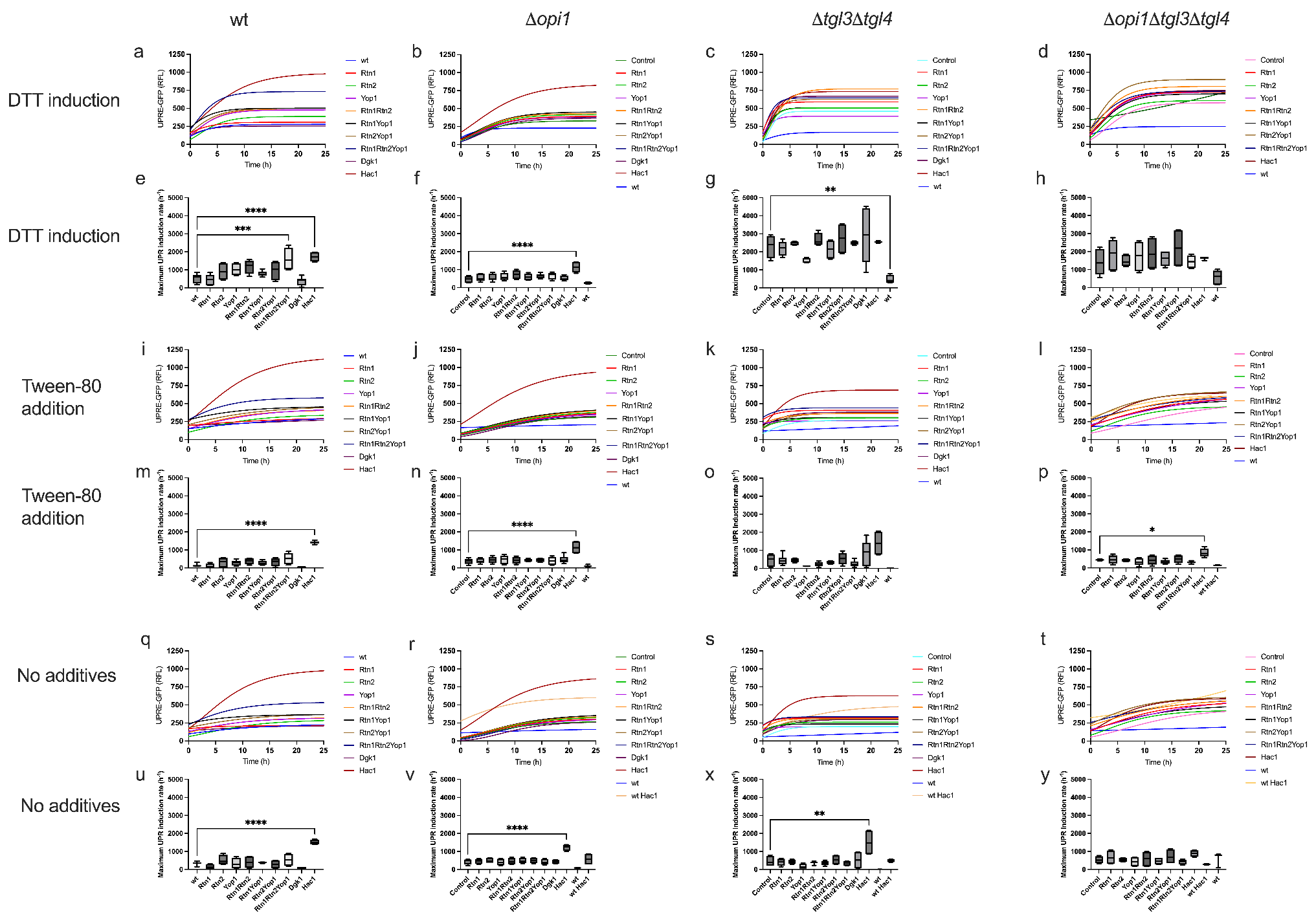


Supplementary figure S3. The UPR and maximum UPR induction rate of the modified strains and corresponding controls under the influence of DTT and Tween-80 and without any UPR inducing additives. Panels a – d show the measured GFP levels and panels e – h the maximum UPR induction rates with DTT induction. Panels i – l show the measured GFP levels and panels m – p the maximum UPR induction rates with Tween-80 induction. Panels q – t show the measured GFP levels and panels u – y the maximum UPR induction rates without any induction. Panels a, e, i, m, q, and u show the results of the WT strain background. Panels b, f, j, n, r, and v show the results of the *Δopi1* strain background. Panels c, g, k, o, s, and x present the results of the *Δtgl3Δtgl4* strain background. Panels d, h, l, p, t, and y show the *Δopi1Δtgl3Δtgl4.* The boxplots show the distribution of the biological replicates of the maximum UPR induction rate. Whiskers extend to the minimum and maximum values and the central line represents the median. P-values were calculated by pairwise comparison to the corresponding control *: P < 0.05; **: P < 0.01; ***: P < 0.001, ****: P < 0.0001.

**References**

1. Niemelä, L. R. K., Koskela, E. V., & Frey, A. D. (2024). Modification of the endoplasmic reticulum morphology enables improved recombinant antibody expression in Saccharomyces cerevisiae. *Journal of Biotechnology*, *387*, 1–11. https://doi.org/10.1016/j.jbiotec.2024.03.009

2. de Ruijter, J. C., Koskela, E. V., & Frey, A. D. (2016). Enhancing antibody folding and secretion by tailoring the Saccharomyces cerevisiae endoplasmic reticulum. *Microbial Cell Factories*, *15*(1), 1–18. https://doi.org/10.1186/s12934-016-0488-5

3. Pincus, D., Chevalier M.W., Aragón, T., van Anken, E., Vidal, S. E., & El-Samad, H. (2010). BiP Binding to the ER-Stress Sensor Ire1 Tunes the Homeostatic Behavior of the Unfolded Protein Response. *PLoS Biology*, *8*(7), e1000415. https://doi.org/10.1371/journal.pbio.1000415

4. Hegemann, J. H., & Heick, S. B. (2011). Delete and Repeat: A Comprehensive Toolkit for Sequential Gene Knockout in the Budding Yeast Saccharomyces cerevisiae. In *Methods in Molecular Biology* (Vol. 765). https://doi.org/10.1007/978-1-61779-197-0_12

5. Valkonen, M., Ward, M., Wang, H., Penttilä, M., & Saloheimo, M. (2003). Improvement of Foreign-Protein Production in Aspergillus niger var. awamori by Constitutive Induction of the Unfolded-Protein Response. *Applied and Environmental Microbiology*, *69*(12). https://doi.org/10.1128/AEM.69.12.6979-6986.2003

6. Kurat, C. F., Natter, K., Petschnigg, J., Wolinski, H., Scheuringer, K., Scholz, H., Zimmermann, R., Leber, R., Zechner, R., & Kohlwein, S. D. (2006). Obese yeast: Triglyceride lipolysis is functionally conserved from mammals to yeast. *Journal of Biological Chemistry*, *281*(1). https://doi.org/10.1074/jbc.M508414200

7. Han, G. S., O’Hara, L., Siniossoglou, S., & Carman, G. M. (2008). Characterization of the yeast DGK1-encoded CTP-dependent diacylglycerol kinase. *Journal of Biological Chemistry*, *283*(29). https://doi.org/10.1074/jbc.M802866200

8. Voeltz, G. K., Prinz, W. A., Shibata, Y., Rist, J. M., & Rapoport, T. A. (2006). A class of membrane proteins shaping the tubular endoplasmic reticulum. *Cell*, *124*(3). https://doi.org/10.1016/j.cell.2005.11.047

9. Schuck, S., Prinz, W. A., Thorn, K. S., Voss, C., & Walter, P. (2009). Membrane expansion alleviates endoplasmic reticulum stress independently of the unfolded protein response. *Journal of Cell Biology*, *187*(4). https://doi.org/10.1083/jcb.200907074

10. Han, G. S., O’Hara, L., Carman, G. M., & Siniossoglou, S. (2008). An unconventional diacylglycerol kinase that regulates phospholipid synthesis and nuclear membrane growth. *Journal of Biological Chemistry*, *283*(29). https://doi.org/10.1074/jbc.M802903200
